# Supplementary material for: Effects of brain training on brain blood flow (The Cognition and Flow Study—CogFlowS): protocol for a feasibility randomised controlled trial of cognitive training in dementia
Source: BMJ Open. 2019 May 22;9(5):e027817. doi: 10.1136/bmjopen-2018-027817 (PMC6538045; doi:10.1136/bmjopen-2018-027817)
Supplement: Supplementary data [file bmjopen-2018-027817supp001.pdf]

Supplementary information. Interview and focus group schedule of questions.

Research Questions

1. What are the barriers to brain training in patients with dementia?
2. What are the facilitators (benefits) to brain training in patients with dementia?
3. Could brain training programs be adapted further to support the participation of patients with dementia?
4. Are there any additional benefits to brain training programs not measured by traditional methods as perceived by the patients and their carers?
5. To explore the lived experience of the patient and their carer and the impact brain training has on them and their life

Section 1. Evaluating the Brain Training Program/ Study Design

- How did participants/carers find the program? (opening question)
- What were the positive aspects to completing the program? (RQ2/4)
- What were the negative aspects to completing the program? (RQ1)
- Did any issues or difficulties arise during the program and how did you manage these? (RQ1/2)
- Do you have any suggestions to improve the program for future studies? (RQ 3)

Section 2. Feasibility (RQ3/4)

- Was it difficult to achieve the minimum number of sessions required to complete the program and if yes, why?
- Were the study assessments the right duration?
- Was the frequency of study visits acceptable?
- Would you make any changes to the study visits to make the experience better for people with dementia?

Section 3. Impact on patients and their carers

- How did the program affect your day to day life? (RQ5)
- Did the frequency or duration of the training interfere with your day to day life? (RQ4)
- How do you feel the brain training program affected your mood? (RQ4)
- How do you feel the brain training program affected your memory? (RQ4)
- How do you feel the brain training program affected your ability to carry out day to day activates? (RQ4)

Section 4. The health belief model

1) Risk susceptibility
    a. How likely do you think you are to develop dementia? (healthy volunteers, MCI)
    b. How do you think undertaking a brain training program affects your risk of developing dementia? (HV, MCI)
2) Risk severity

   a. If you were to develop dementia, how serious do you think the consequences would be for you? (HV, MCI)
   b. How severe do you think your memory difficulties are? (AzD, MCI)
   c. How do you feel the brain training program has affected the severity of your symptoms? (AzD, MCI)

3) Benefits to action
   a. What helped you to complete the brain training program? (RQ2)
4) Barriers to action
   a. What stopped you with you completing the brain training program? (RQ1)
5) Self-efficacy
   a. If you experienced difficulties, how did you overcome these to complete the program? (RQ1/2)
6) Cues to action
   a. Did anything support or prompt you to complete the program? (RQ1/2)
   b. Did any thoughts, feelings or symptoms affect you completing the program? (RQ1/2)

Data Security and Storage

Source documents are original documents, data, and records from which participants' CRF data are obtained. These include, but are not limited to, hospital records (from which medical history and previous and concurrent medication may be summarised into the CRF), clinical and office charts, laboratory and pharmacy records, radiographs/scan reports, and correspondence, interview and focus group recordings and transcripts.

CRF entries will be considered source data if the CRF is the site of the original recording (e.g., there is no other written or electronic record of data). In this study the CRF will be used as the source document for; cognition (ACE-III), mood (GDS-15), function/ADLs (Lawton-IADL), quality of life (DEMQOL), handedness (Edinburgh Handedness Inventory), and assessments of neurovascular function (TCD), blood pressure, heart rate and $ETCO_2$.

All documents will be stored safely in confidential conditions. On all study-specific documents, other than the signed consent, the participant will be referred to by the study participant number/code, not by name. All parameters (signals) that are collected during the measurement will be saved using a coded filename. The name and other identifying detail will NOT be included in any study data electronic file. All study data will be entered into Microsoft Excel for Windows. All files will be encrypted and stored on a password secured computer/laptop, which will have restricted access to members who are authorised on the authorisation log. All paper documentation will be stored in a locked filing cabinet, in a locked office, on University of Leicester premises that require identification to enter the building. The participants will be identified by a study specific participant number and/or code in any database. The name and any other identifying detail will NOT be included in any study data electronic file.
