# Supplementary material for: Effects of brain training on brain blood flow (The Cognition and Flow Study—CogFlowS): protocol for a feasibility randomised controlled trial of cognitive training in dementia
Source: BMJ Open. 2019 May 22;9(5):e027817. doi: 10.1136/bmjopen-2018-027817 (PMC6538045; doi:10.1136/bmjopen-2018-027817)
Supplement: Author's manuscript [file bmjopen-2018-027817.draft_revisions.pdf]

BMJ Open is committed to open peer review. As part of this commitment we make the peer review history of every article we publish publicly available.

When an article is published we post the peer reviewers' comments and the authors' responses online. We also post the versions of the paper that were used during peer review. These are the versions that the peer review comments apply to.

The versions of the paper that follow are the versions that were submitted during the peer review process. They are not the versions of record or the final published versions. They should not be cited or distributed as the published version of this manuscript.

BMJ Open is an open access journal and the full, final, typeset and author-corrected version of record of the manuscript is available on our site with no access controls, subscription charges or pay-per-view fees (http://bmjopen.bmj.com).

If you have any questions on BMJ Open's open peer review process please email info.bmjopen@bmj.com

The Effects of Brain Training on Brain Blood Flow (The Cognition and Flow Study -CogFlowS): A Protocol for A Feasibility Randomised Controlled Trial of Cognitive Training in Dementia.

Journal:	*BMJ Open*
Manuscript ID	bmjopen-2018-027817
Article Type:	Protocol
Date Submitted by the Author:	08-Nov-2018

Complete List of Authors:	Beishon, Lucy; University of Leicester College of Medicine Biological Sciences and Psychology,<br>Panerai, Ronney<br>Evley, Rachel<br>Subramaniam, Hari<br>Mukaetova-Ladinska, Elizabeta<br>Robinson, Thompson; University of Leicester College of Medicine Biological Sciences and Psychology, Cardiovascular Sciences<br>Haunton, Victoria

Keywords:	Neuroimaging, Cerebral haemodynamics, Alzheimer's disease, Mild cognitive impairment, Transcranial Doppler ultrasonography, cognitive impairment

SCHOLARONE™
Manuscripts

Title

The Effects of Brain Training on Brain Blood Flow (The Cognition and Flow Study -CogFlowS): A Protocol for A Feasibility Randomised Controlled Trial of Cognitive Training in Dementia.

Authors

Lucy Beishon[a], Ronney B Panerai[a,b], Rachel Evley[c], Hari Subramaniam[d], Elizabeta Mukaetova-Ladinska[d,e], Thompson G Robinson[a,b], Victoria J Haunton[a,b].

Affiliations

[a] University of Leicester, Department of Cardiovascular Sciences, Leicester, UK

[b] NIHR Leicester Biomedical Research Centre, British Heart Foundation Cardiovascular Research Centre, Glenfield Hospital, Leicester, UK

[c] Department of Clinical Neurosciences, Nottingham, UK

[d] The Evington Centre, Leicestershire Partnership NHS Trust, Leicester, UK

[e] University of Leicester, Department of Neuroscience, Psychology and Behaviour, Leicester, UK

Corresponding Author

Lucy Beishon

Lb330@le.ac.uk

Room 228, Level 2 Clinical Sciences Building

Leicester Royal Infirmary

Leicester

LE2 7LX

For peer review only

For peer review only - http://bmjopen.bmj.com/site/about/guidelines.xhtml

Tel: 0116 252 3134

Key words

Cognitive impairment

Neuroimaging

Cerebral haemodynamics

Alzheimer's disease

Mild cognitive impairment

Transcranial Doppler ultrasonography

Word count

3679

Protocol date and version number

30/10/2018, version 2

Trial sponsor

Dr Michelle Meussel

Research Governance Office, Leicester General Hospital

Leicester

LE5 4PW

01162584077

uolsponsor@le.ac.uk

For peer review only

Abstract

Introduction

Cognitive training is an emerging non-pharmacological treatment to improve cognitive and physical function in mild cognitive impairment (MCI) and early Alzheimer's disease (AD). Abnormal brain blood flow is a key process in the development of cognitive decline. However, no studies have explored the effects of cognitive training on brain blood flow in dementia. The primary aim of this study is to assess the feasibility for a large scale, randomised controlled trial of cognitive training in healthy older adults (HC), MCI, and early AD.

Methods and analysis

This study will recruit 60 participants, in three sub-groups of twenty (MCI, HC, AD), from primary, secondary, and community services. Participants will be randomised to an eight to twelve week computerised cognitive training programme (five x 30 minute sessions per week), or waiting-list control. Participants will undergo baseline and follow-up assessments of: mood, cognition, quality of life, and activities of daily living. Cerebral blood flow will be measured at rest and during task activation (pre- and post-training) by bilateral transcranial Doppler ultrasonography, alongside heart rate (3-lead ECG), end-tidal CO_2 (capnography), and beat-to-beat blood pressure (Finometer). Participants will be offered to join a focus group or semi-structured interview to explore barriers and facilitators to cognitive training in patients with dementia. Qualitative data will be analysed using framework analysis, and data will be integrated using mixed methods matrices.

Ethics and dissemination

Bradford Leeds Research Ethics committee awarded a favorable opinion (18/YH/0396). Results of the study will be published in peer reviewed journals, and presented at national and international conferences on ageing and dementia.

For peer review only

Registration

ClinicalTrials.Gov unique identifier: NCT03656107.

Article summary: Strengths and limitations (5 bullet points)

- This study uses a mixed methods approach to investigate the feasibility of cognitive training in healthy older adults, mild cognitive impairment, and Alzheimer's disease
- This study includes an assessment of the effects of cognitive training on neurovascular function, by transcranial Doppler ultrasonography
- This study includes a broad range of outcome measures to take a holistic approach to evaluating cognitive training in dementia
- The primary aim of this study is to identify the feasibility of this protocol in patients with cognitive impairment, and as such is not a fully powered trial
- This study is limited to identifying these outcomes in healthy older adults, mild cognitive impairment, and Alzheimer's disease with mild to moderate deficits

For peer review only

For peer review only - http://bmjopen.bmj.com/site/about/guidelines.xhtml

Introduction

The incidence of dementia is rising as the population ages, with 46.8 million people living with dementia worldwide (1, 2). By 2030, this is projected to rise to 75 million (1, 2). However, there are currently few diagnostic or therapeutic strategies available to offer these patients. Dementia is a progressive condition characterised by gradual loss of cognitive or non-cognitive higher functions (i.e. language, visuospatial, attention) (3). Mild cognitive impairment (MCI) is characterised by subjective and objective decline in cognitive function, but with preserved functional independence in daily living (4). It has become increasingly recognised that deranged vascular function is an early contributor to the deposition of amyloid plaques and tau tangles in Alzheimer's disease (AD), and that these pathologies exacerbate one another (two-hit hypothesis) (5). In a recent systematic review and meta-analysis, we demonstrated clear abnormalities in vascular function across a number of imaging modalities at the MCI stage (6). Thus, treatments that can improve cerebral perfusion or vascular function could represent an early treatment option for dementia (6). In this study, the target populations are: healthy older adults (as controls), adults with AD, and MCI, to capture the effects of cognitive training on vascular physiology at an early stage in the dementia process.

The Cognition and Flow Study (CogFlowS) is a feasibility, parallel, randomised controlled trial (RCT) to examine the use of a cognitive training programme in adults with MCI, early AD and healthy older adults (HC). In addition to measuring cognitive and functional outcomes, this study will also use transcranial Doppler ultrasonography (TCD) to measure changes in cerebral blood flow (CBF) before and after an 8-12-week cognitive training program.

Cognitive interventions can be considered as three broad categories: cognitive training, cognitive rehabilitation and cognitive stimulation (7, 8). Cognitive training describes a structured, guided program of standardised tasks designed to provide practice or training with a specified cognitive domain(s), to translate into functional benefits (7, 8). Cognitive training is attractive in that it offers a cost effective, non-invasive and acceptable intervention to patients, with no reported adverse effects

For peer review only - http://bmjopen.bmj.com/site/about/guidelines.xhtml

(9). However, high quality RCT evidence is lacking in studies of cognitive training in dementia (8, 10-12). Two previous Cochrane reviews (8, 10), highlighted that the evidence was sparse, with many of the studies under-powered, of low-to-moderate quality, and with relatively few for MCI (8, 10). Recent systematic reviews of cognitive training in MCI have demonstrated moderate benefits in verbal learning and memory, global cognition, non-verbal learning, working memory, attention and psychosocial functioning (11, 13). Thus far, studies of cognitive training in dementia have been heterogeneous in study design, participants included, type and definition of training, outcome measures, and duration and intensity of intervention (8, 10, 12, 14, 15). This hampers the adequate meta-analysis of data, and of conclusions that can be drawn from pooled analyses (12, 16). Few studies have included neuroimaging outcomes to explore brain plasticity or neural mechanisms underlying changes in cognition after cognitive training (16). Therefore, the CogFlowS study not only addresses the development of a future high quality RCT of cognitive training in dementia, but also aims to fill the evidence gap on the use of TCD to assess neurovascular plasticity.

TCD is a non-invasive, ultrasound based imaging modality that allows measurements of CBF velocity (CBFv) at rest (17) and following activation by cognitive tasks (18). TCD has a number of advantages over other functional imaging techniques (magnetic resonance imaging (MRI) and positron emission tomography (PET), including lower cost, portability, relative ease of operator training, high temporal resolution and higher acceptability to patients (19, 20). TCD is particularly suited to older people, and those with dementia, as it can be used in patients with pacemakers, metal implants and claustrophobia (19). The use of TCD is a novel method to investigate neurovascular training effects and for markers predictive of which patients may benefit from particular cognitive training programmes. This would allow the development of a personalised approach to developing cognitive training programmes for people with dementia.

The use of neuroimaging outcomes has also been limited in cognitive training studies (16, 21), but understanding the training effects occurring at the physiological level may provide important mechanistic information to any potential benefits conferred by cognitive training (22-24). Belleville et

For peer review only - http://bmjopen.bmj.com/site/about/guidelines.xhtml

al demonstrated increased parietal activation in patients with MCI, following a multi-modal cognitive training program designed to improve episodic memory (22). In a near-infrared spectroscopy study by Vermeij et al, patients with MCI had greater training benefits at high load working memory where participants had greater pre-frontal activation (25), whereas baseline global and hippocampal atrophy were predictive of poorer training gains in healthy older adults and those with MCI (23). Therefore, imaging biomarkers can potentially be used to predict and tailor interventions to individual patient's needs (23, 24). Whilst a number of studies have demonstrated positive structural and functional effects of cognitive training (24, 26, 27), they have largely focused on healthy older adults (26), with the use of structural (26) or functional MRI (22, 23) and PET (28, 29); there have been no TCD studies examining changes in neurovascular response to cognitive training in patients with cognitive impairment. In addition to the inclusion of neuroimaging, this study utilises a mixed methods approach. This study includes a qualitative component to explore any benefits, problems or barriers to engaging with the training program, and to identify any benefits not measured by traditional outcomes.

The primary objective for this study is to determine the feasibility for a large scale RCT of cognitive training in HC, AD, and MCI. Secondary objectives seek to identify any clinical benefits of a cognitive training program in terms of: activities of daily living, cognition, mood, and quality of life, and TCD-measured task activation responses in brain blood flow. In addition, the qualitative study seeks to explore barriers and facilitators to the cognitive training programme, how programs can be adapted to support participation for patients living with dementia, and any additional benefits not measured through traditional methods as perceived by patients and their carers. Finally, we plan to explore the lived experience of the patient and their carer, and the impact cognitive training has on them and their life.

For peer review only - http://bmjopen.bmj.com/site/about/guidelines.xhtml

Methods and analysis

This protocol was reported in accordance with the SPRIT checklist (30), which is included in Supplementary information.

Sample selection

Healthy volunteers will be recruited through poster advertisement, friends, family, and carers of enrolled patient participants, Join Dementia Research, and the research interested list at the University of Leicester. Participants with a diagnosis of MCI, or AD, will be recruited through poster advertisement, secondary care services (outpatient clinics within the University Hospitals of Leicester (acute) and the Leicestershire Partnership (mental health) NHS Trusts), research interested lists, Join Dementia Research, and local community groups. In addition, participants will be sought through letter invitation from their GP surgery. Participants will be allowed a minimum of 24 hours to decide if they would like to enrol in the study, and to contact the researcher or research delivery team at the Leicestershire Partnership Trust, who will arrange for them to undergo an eligibility assessment, formal consent, and study enrolment. As part of the eligibility assessment participants will undergo a screening cognitive assessment (Montreal Cognitive Assessment – MoCA) to ensure deficits are classified as mild to moderate. Participants will be consented for this, and the MoCA will not form part of the baseline assessments. Flow charts of the recruitment process and participant procedures can be seen in Figures 1 and 2, respectively.

The planned recruitment target for this study is 60 participants, divided into three sub-groups of twenty (healthy older adults, AD, and MCI). As the primary aim of this study is feasibility, a formal sample size calculation has not been performed. Recruiting between 24 and 50 participants for a feasibility study is acceptable and therefore the planned target of 60 participants is sufficiently large enough whilst giving a margin for drop-outs and loss to follow-up (31, 32).

For peer review only

For peer review only - http://bmjopen.bmj.com/site/about/guidelines.xhtml

The following inclusion and exclusion criteria were designed to identify a sample of participants that will be able to engage with a cognitive training programme, and to exclude any major medical co-morbidities that can affect CBF:

Inclusion criteria

1) Healthy controls will be free of any medical co-morbidity or medication that could adversely affect cognition. Volunteers with well-controlled co-morbidities (i.e. hypertension, diabetes, will be considered for inclusion)
2) MCI as defined by NIA/AA 2011 (33) and Petersen criteria (4)
3) AD as defined by the NIA/AA 2011 criteria (33)

   And:

4) Deficits will be mild to moderate as defined by Montreal Cognitive Assessment (MoCA) score of 19-26 for MCI, and 9-18 for AD (34-36).
5) Willing to participate
6) Capacity to consent to the study/personal consultee
7) Patients on and off anti-dementia medications will be included (acetylcholinesterase inhibitors, glutamate receptor antagonists)
8) Good understanding of written and spoken English
9) Age >50 years
10) Access to the internet and a computer/laptop device.

Exclusion criteria

1) Healthy controls with any medical co-morbidity or medication that could adversely affect cognition, or poorly controlled medical co-morbidities (i.e. hypertension, diabetes)

For peer review only - http://bmjopen.bmj.com/site/about/guidelines.xhtml

2) Unwilling to take part

3) Unable to consent/no personal consultee

4) Major medical co-morbidity: severe heart failure (ejection fraction <20%), carotid artery stenosis, severe respiratory disease, major stroke

5) Pregnancy, planning pregnancy, or lactating

6) Inadequate bilateral TCD windows

7) Participants already enrolled into other interventional studies that would confound study results

8) Insufficient understanding of written and spoken English

9) Age <50 years

10) No access to the internet and a computer/laptop device

Intervention

Participants randomised to the intervention will complete an eight to twelve week cognitive training programme. Participants will be required to complete five, 30 minute sessions per week for eight to twelve weeks. The cognitive training programme will be provided by Lumosity© as part of a collaboration through the Human Cognition Project. Lumosity© is commercially available software, developed by a group of neuropsychologists, which has been used across several studies of brain training and disciplines (37-39). These studies have been included in a number of systematic reviews and meta-analysis (11, 40). It is a multi-domain, online-based cognitive training tool, which is relatively easy to use and administer. It has been designed to adapt to the individual's cognitive function in order to personalise the training program to their needs. Exercises will be selected with the support of Lumosity© to target the following cognitive domains: attention, memory, visuospatial, verbal fluency, and language. Compliance will be monitored through Lumosity© online software which will log and track the number of minutes and times per week a participant has spent training, in order to calculate the dose of training to which each participant has been exposed.

For peer review only - http://bmjopen.bmj.com/site/about/guidelines.xhtml

For peer review only

Randomisation

Randomisation will be performed using Sealed Envelope©, by the researcher. This is an online-based randomisation tool which uses random permuted blocks to allocate participants to waiting list control or intervention. Participants will be enrolled and assigned a PIN consecutively, and randomised to a treatment arm corresponding to the PIN. Given the nature of the trial, it is not possible to blind participants to the intervention. The Investigator will be providing weekly telephone support for the intervention group, in addition to undertaking baseline and follow-up measurements, and therefore blinding of the investigator is also not possible. However, data analysis will be blinded by generating a batch-anonymised data set. Randomisation will be undertaken at the initial visit. Once participants have been randomised to the intervention arm, they will be provided with information on how to access and use the cognitive training programme at home.

Data collection and integration

All participants (intervention and control groups) will be required to attend two assessments: baseline, and follow-up at eight to twelve weeks post-training. Assessments will be carried out at the participant's home, or designated research space at the Leicestershire Partnership Trust, or Cerebral Haemodynamics in Ageing and Stroke Medicine (CHiASM) research space. Data will be collected on demographics, medical history, and medication use. Assessments of: cognition [Addenbrooke's cognitive examination III (ACE-III)], mood (Geriatric Depression Scale 15), activities of daily living (Lawton instrumental activities of daily living), and quality of life (Dementia Rated Quality of Life), will be carried out at both visits. An assessment of neurovascular function will be carried out at the CHiASM research space, a temperature controlled room, free of noise and distraction. Participants will be seated throughout the protocol. All measurements will be made at rest (five minute baseline recording), and during cerebral activation (selected cognitive tasks). CBFv will be measured continuously by insonation of the bilateral middle cerebral arteries (MCA) (DWL Doppler Box), using 2 MHz probes, fixed in place with a headframe. Beat-to-beat blood pressure will be measured

For peer review only - http://bmjopen.bmj.com/site/about/guidelines.xhtml

continuously using a Finometer cuff on the non-dominant middle finger (Finapres Medical Systems, Amsterdam), and calibrated using a brachial blood pressure recording (UA767 BP monitor). End-tidal CO_2 will be measured by capnography using nasal cannulae (Capnochek Plus), and the R-R interval will be recorded using a 3-lead ECG. The task activation protocol has been published previously (41, 42). In brief, participants will be presented with five tasks selected from the ACE-III, and percentage increase in CBFv from resting baseline will be calculated at task initiation. The tasks will be selected for five cognitive domains: attention, memory, visuospatial, language, and verbal fluency, based on recent studies conducted by this group (42, 43).

At least one week after completion of the cognitive training programme, participants with a diagnosis of MCI, or AD, will be invited to attend a semi-structured interview or focus group. The schedule of questions can be seen in supplementary information. The schedule of questions has been framed around the six constructs of the health belief model (risk susceptibility, risk severity, benefits to action, barriers to action, self-efficacy, and cues to action) (44) to evaluate the cognitive training programme, and the experience of the participant and their carer. Interviews and focus groups will be recorded continuously with a digital audio recorder, and notes of non-verbal and paralinguistic clues will be made. All digital recordings will be transcribed within five to seven days of the interview or focus group. Semi-structured interviews are an iterative process, following the first couple of interviews the transcripts will be analysed and the themes and concepts emerging from these initial data will be further explored at the following interviews.

Data security and storage will be conducted in line with general data protection regulations (https://eugdpr.org/), and details regarding data security and storage can be seen in Supplementary information.

Trial discontinuation

For peer review only - http://bmjopen.bmj.com/site/about/guidelines.xhtml

For peer review only

This is not a drug intervention trial, and therefore interim analysis will not be required. Criteria for trial discontinuation are as follows: ineligibility, significant protocol deviation, significant non-adherence to the programme, an adverse event or disease progression resulting in inability to comply with study procedures, consent withdrawn, or lost to follow-up.

Data analysis and interpretation

The difference for each participant between baseline and follow-up assessment for each of the continuous outcome measures described above will be reported along with the overall mean difference.

CBFv data will be recorded and stored in the PHYSIDAS system, and analysed offline. Data will be visually inspected for quality, and files rejected where quality is deemed to be poor, with reasons. Peak percentage change in CBFv will be calculated relative to a twenty second baseline, prior to task initiation.

The findings from the semi-structured interviews and focus groups will be evaluated using framework analysis (41). The digital recordings of the interviews and focus groups will be transcribed verbatim, the transcripts will be read in detail, i.e. line-by-line, and open codes will be formed categorising and conceptualising the responses and identifying the major themes. Two researchers LB & RE will independently code the first few transcripts to ensure consistency in coding.

Following this initial coding the analytical framework will be developed, this is an iterative process and will develop through coding of additional transcripts. Once the final transcript has been coded the analytic framework will be used to generate the framework matrix. The framework matrix will be developed in NVivo 11 (QSR International), and allow for the recognition of patterns and outliers within the data. Respondent validation will be used to establish a degree of correspondence between the researcher's views and those of the research participant (45).

For peer review only - http://bmjopen.bmj.com/site/about/guidelines.xhtml

Data from the quantitative and qualitative arms of the study will be integrated using mixed methods approaches of triangulation and mixed methods matrices (46).

Statistical analysis and sample size calculation

Data will be checked for normality, and non-normally distributed data will be appropriately transformed, and parametric tests applied. Data will be reported as mean (standard deviation) for continuous variables, and number (percentage) for nominal data. Differences between the intervention and control groups will be analysed within population group (HC/AD/MCI) using Chi-Square for nominal data, and independent t-testing for continuous data. Any differences between population groups will also be reported. To assess the impact of the intervention and population group on each of these differences, a two-way ANOVA will be carried out. The interaction between population group and treatment group will be analysed to assess if the difference between the treatment groups is different between the population groups. Data will be recorded in Microsoft Excel. Statistical analyses will be performed using the latest version of SPSS for Windows (SPSS v.24), and graphs will be produced using the latest version of GraphPad Prism for Windows.

Study limitations

This is a feasibility study, and thus will not be powered adequately to detect changes in the secondary end points outlined above. However, given the novel aspects of this study protocol, such as the inclusion of a TCD-measured changes in CBF responses, and the mixed methods design, this requires evaluation of the feasibility before moving to a larger, fully powered trial of cognitive training in dementia. Furthermore, this study will include assessments of patient experience and tolerability to facilitate this. Although TCD affords excellent temporal resolution in the measurement of CBFv, the MCA supplies blood to approximately 80% of the cerebral cortex, and therefore reflects a global measure of perfusion, and cannot discriminate where changes are occurring, due to poor spatial resolution. Techniques such as MRI, and PET scans afford better spatial resolution, but have their own limitations, particularly in older patients with cognitive deficits. TCD-measured CBFv relies on the

For peer review only - http://bmjopen.bmj.com/site/about/guidelines.xhtml

For peer review only

assumption that the vessel diameter remains constant, despite fluctuations in CO_2 and blood pressure (17). This study elected to focus on healthy older adults, MCI and AD in the first instance. Deficits in CBFv have been demonstrated in both MCI and AD, but inclusion of participants with vascular cognitive impairment would provide further information on the capacity for plasticity to neurovascular physiology amongst dementia sub-types. In addition, only participants with mild to moderate deficits will be included, given that data thus far do not support the use of cognitive training in more advanced dementia (10, 11).

Ethics and dissemination

Ethics and safety considerations

This study has been reviewed by the Bradford Leeds Research Ethics Committee (REC), and was granted a favorable opinion on the 7/11/18 (18/YH/0396). All participants with capacity in this study will provide informed, written consent, and the study will be conducted in accordance with the Declaration of Helsinki and Good Medical Practice guidelines (47). Given the nature of dementia, not all participants will have capacity to consent to the study. In this instance, a personal consultee (friend, relative, or carer) will be consulted for their opinion on whether the participant is likely to have wanted to participate in this study, and they will be asked to sign a consultee declaration form. It was not considered ethical to exclude participants on the grounds of capacity, given the nature of the disease studied.

Dissemination

The results from this study will be submitted to peer-reviewed journals in ageing and dementia research, and presented at national and international conferences on these themes. We anticipate the results will be presented and published in late 2020, early 2021. In addition, the results will be disseminated to members of the community through the Alzheimer's Society, community groups, podcasts, and social media.

For peer review only - http://bmjopen.bmj.com/site/about/guidelines.xhtml

For peer review only

Trial registration

This study has been registered on ClinicalTrials.gov (ref: NCT03656107). The trial was registered prior to initiation, and will be completed within six weeks of recruitment of the first study participant.

Ethics approval

Ethical approval was obtained from the Bradford Leeds Research Ethics Committee (18/YH/0396)

Competing interests

None.

Author Contributions

The quantitative arm of the study was conceived and designed by LB, VJH, RBP, and TGR. LB and RE designed the qualitative arm of the study. HS and EML reviewed and edited the protocol. All authors assisted in drafting this manuscript.

Trial committees and monitoring

The study will be monitored at six-monthly intervals throughout its duration by a trial steering committee, independent of the funder, consisting of lay and independent academic members. Each site will have a lead investigator who is responsible for the identification and recruitment of participants, data collection and completion for CRFs, follow-up measurements, and adherence to study protocols. Serious adverse events (SAEs) will be reported to the sponsor within one working day of identification, and the Sponsor will report all serious unexpected or adverse reactions to the ethics committee concerned. The chief investigator will submit an annual report to the ethics committee which lists all SAEs. Where necessary, protocol amendments will be submitted to the study sponsor or ethics committee for approval.

Provenance and peer review

Not commissioned.

For peer review only - http://bmjopen.bmj.com/site/about/guidelines.xhtml

Data sharing agreement

The data will be stored in the Leicester Research Archive after study completion, and data will be made available at request.

Funding

This study was reviewed and funded by the Dunhill Medical Trust, and LB is a Dunhill research training fellow (RTF1806\27). TGR is an NIHR Senior Investigator. This funding source had and will not have a role in the design, conduct, analyses, interpretation of the data, or decision to submit results. The cognitive training programme is being provided by Lumosity© free of charge, as part of an industry-institution collaboration through the Human Cognition Project. Lumosity© is providing advice and support on the composition and nature of the programme. However, this study is financially independent of Lumosity©, and Lumosity© cannot influence or change the results or outcomes of this study.

For peer review only

For peer review only - http://bmjopen.bmj.com/site/about/guidelines.xhtml

References

1. Alzheimer's-society. Demography: Alzheimer's Society; 2016 [updated 2016-11-28. Available from: https://www.alzheimers.org.uk/info/20091/position_statements/93/demography.
2. Alzheimer's-Association. 2014 Alzheimer's disease facts and figures. Alzheimers Dement. 2014;10(2):e47-92.
3. Robinson L, Tang E, Taylor JP. Dementia: timely diagnosis and early intervention. Bmj. 2015;350:h3029.
4. Petersen RC. Mild cognitive impairment as a diagnostic entity. J Intern Med. 2004;256(3):183-94.
5. Zlokovic BV. Neurovascular pathways to neurodegeneration in Alzheimer's disease and other disorders. Nat Rev Neurosci. 2011;12(12):723-38.
6. Beishon L, Haunton VJ, Panerai RB, Robinson TG. Cerebral Hemodynamics in Mild Cognitive Impairment: A Systematic Review. J Alzheimers Dis. 2017;59(1):369-85.
7. Belleville S. Cognitive training for persons with mild cognitive impairment. Int Psychogeriatr. 2008;20(1):57-66.
8. Martin M, Clare L, Altgassen AM, Cameron MH, Zehnder F. Cognition-based interventions for healthy older people and people with mild cognitive impairment. Cochrane Database Syst Rev. 2011(1):Cd006220.
9. Gates NJ, Sachdev P. Is cognitive training an effective treatment for preclinical and early Alzheimer's disease? J Alzheimers Dis. 2014;42 Suppl 4:S551-9.
10. Bahar-Fuchs A, Clare L, Woods B. Cognitive training and cognitive rehabilitation for persons with mild to moderate dementia of the Alzheimer's or vascular type: a review. Alzheimers Res Ther. 2013;5(4):35.
11. Hill NT, Mowszowski L, Naismith SL, Chadwick VL, Valenzuela M, Lampit A. Computerized Cognitive Training in Older Adults With Mild Cognitive Impairment or Dementia: A Systematic Review and Meta-Analysis. Am J Psychiatry. 2017;174(4):329-40.
12. Gates NJ, Sachdev PS, Fiatarone Singh MA, Valenzuela M. Cognitive and memory training in adults at risk of dementia: a systematic review. BMC Geriatr. 2011;11:55.
13. Li H, Li J, Li N, Li B, Wang P, Zhou T. Cognitive intervention for persons with mild cognitive impairment: A meta-analysis. Ageing Res Rev. 2011;10(2):285-96.
14. Huntley JD, Gould RL, Liu K, Smith M, Howard RJ. Do cognitive interventions improve general cognition in dementia? A meta-analysis and meta-regression. BMJ Open. 2015;5(4).
15. Reijnders J, van Heugten C, van Boxtel M. Cognitive interventions in healthy older adults and people with mild cognitive impairment: a systematic review. Ageing Res Rev. 2013;12(1):263-75.
16. Coyle H, Traynor V, Solowij N. Computerized and virtual reality cognitive training for individuals at high risk of cognitive decline: systematic review of the literature. Am J Geriatr Psychiatry. 2015;23(4):335-59.
17. Panerai RB. Transcranial Doppler for evaluation of cerebral autoregulation. Clin Auton Res. 2009;19(4):197-211.
18. Stroobant N, Vingerhoets G. Transcranial Doppler ultrasonography monitoring of cerebral hemodynamics during performance of cognitive tasks: a review. Neuropsychol Rev. 2000;10(4):213-31.
19. van Beek AH, Claassen JA, Rikkert MG, Jansen RW. Cerebral autoregulation: an overview of current concepts and methodology with special focus on the elderly. J Cereb Blood Flow Metab. 2008;28(6):1071-85.
20. Tomek A, Urbanova B, Hort J. Utility of transcranial ultrasound in predicting Alzheimer's disease risk. J Alzheimers Dis. 2014;42:S365-S74.
21. Vemuri P, Fields J, Peter J, Klöppel S. Cognitive interventions in Alzheimer's and Parkinson's diseases: emerging mechanisms and role of imaging. Curr Opin Neurol. 2016;29(4):405-11.

For peer review only - http://bmjopen.bmj.com/site/about/guidelines.xhtml

22. Belleville S, Clement F, Mellah S, Gilbert B, Fontaine F, Gauthier S. Training-related brain plasticity in subjects at risk of developing Alzheimer's disease. Brain. 2011;134(Pt 6):1623-34.
23. Vermeij A, Claassen JA, Dautzenberg PL, Kessels RP. Transfer and maintenance effects of online working-memory training in normal ageing and mild cognitive impairment. Neuropsychol Rehabil. 2016;26(5-6):783-809.
24. Belleville S, Bherer L. Biomarkers of Cognitive Training Effects in Aging. Curr Transl Geriatr Exp Gerontol Rep. 2012;1(2):104-10.
25. Vermeij A, Kessels RPC, Heskamp L, Simons EMF, Dautzenberg PLJ, Claassen JAHR. Prefrontal activation may predict working-memory training gain in normal aging and mild cognitive impairment. Brain Imaging and Behavior. 2016:1-14.
26. Engvig A, Fjell AM, Westlye LT, Moberget T, Sundseth O, Larsen VA, et al. Effects of memory training on cortical thickness in the elderly. Neuroimage. 2010;52(4):1667-76.
27. Yang H, Leaver AM, Siddarth P, Paholpak P, Ercoli L, St. Cyr NM, et al. Neurochemical and Neuroanatomical Plasticity Following Memory Training and Yoga Interventions in Older Adults with Mild Cognitive Impairment. Front Aging Neurosci. 2016;8.
28. Ciarmiello A, Gaeta MC, Benso F, Del Sette M. FDG-PET in the Evaluation of Brain Metabolic Changes Induced by Cognitive Stimulation in aMCI Subjects. Curr Radiopharm. 2015;8(1):69-75.
29. Forster S, Buschert VC, Teipel SJ, Friese U, Buchholz HG, Drzezga A, et al. Effects of a 6-month cognitive intervention on brain metabolism in patients with amnestic MCI and mild Alzheimer's disease. J Alzheimers Dis. 2011;26 Suppl 3:337-48.
30. Chan AW, Tetzlaff JM, Altman DG, Laupacis A, Gøtzsche PC, Krleža-Jerić K, et al. SPIRIT 2013 Statement: Defining Standard Protocol Items for Clinical Trials. Ann Intern Med. 2013;158(3):200-7.
31. Sim J, Lewis M. The size of a pilot study for a clinical trial should be calculated in relation to considerations of precision and efficiency. J Clin Epidemiol. 2012;65(3):301-8.
32. Julious SA. Sample sizes for clinical trials with normal data. Stat Med. 2004;23(12):1921-86.
33. Albert MS, DeKosky ST, Dickson D, Dubois B, Feldman HH, Fox NC, et al. The diagnosis of mild cognitive impairment due to Alzheimer's disease: recommendations from the National Institute on Aging-Alzheimer's Association workgroups on diagnostic guidelines for Alzheimer's disease. Alzheimers Dement. 2011;7(3):270-9.
34. Saczynski JS, Inouye SK, Guess J, Jones RN, Fong TG, Nemeth E, et al. The Montreal Cognitive Assessment (MoCA): Creating a Crosswalk with the Mini-Mental State Examination. J Am Geriatr Soc. 2015;63(11):2370-4.
35. Borland E, Nagga K, Nilsson PM, Minthon L, Nilsson ED, Palmqvist S. The Montreal Cognitive Assessment: Normative Data from a Large Swedish Population-Based Cohort. J Alzheimers Dis. 2017;59(3):893-901.
36. Tan JP, Li N, Gao J, Wang LN, Zhao YM, Yu BC, et al. Optimal cutoff scores for dementia and mild cognitive impairment of the Montreal Cognitive Assessment among elderly and oldest-old Chinese population. J Alzheimers Dis. 2015;43(4):1403-12.
37. Kesler S, Hadi Hosseini SM, Heckler C, Janelsins M, Palesh O, Mustian K, et al. Cognitive training for improving executive function in chemotherapy-treated breast cancer survivors. Clin Breast Cancer. 2013;13(4):299-306.
38. Finn M, McDonald S. A single case study of computerised cognitive training for older persons with mild cognitive impairment. NeuroRehabilitation. 2014;35(2):261-70.
39. Ballesteros S, Prieto A, Mayas J, Toril P, Pita C, Ponce de León L, et al. Brain training with non-action video games enhances aspects of cognition in older adults: a randomized controlled trial. Front Aging Neurosci. 2014;6.
40. Lampit A, Hallock H, Valenzuela M. Computerized cognitive training in cognitively healthy older adults: a systematic review and meta-analysis of effect modifiers. PLoS Med. 2014;11(11):e1001756.

For peer review only - http://bmjopen.bmj.com/site/about/guidelines.xhtml

41. Beishon L, Williams CAL, Panerai RB, Robinson TG, Haunton VJ. Reproducibility of task activation using the Addenbrooke's cognitive examination in healthy controls: A functional Transcranial Doppler ultrasonography study. J Neurosci Methods. 2017.

42. Beishon LC, Williams CAL, Panerai RB, Robinson TG, Haunton VJ. The assessment of neurovascular coupling with the Addenbrooke's Cognitive Examination: a functional Transcranial Doppler Ultrasonographic Study. J Neurophysiol. 2017:jn.00698.2017.

43. Beishon L, Panerai RB, Robinson TG, Subramaniam H, Haunton VJ. The assessment of cerebrovascular response to a language task from the Addenbrooke's cognitive examination in cognitive impairment: A feasibility functional transcranial Doppler ultrasonography study. Journal of Alzheimer's Disease Reports. 2018;2:153-64.

44. Jones CL, Jensen JD, Scherr CL, Brown NR, Christy K, Weaver J. The Health Belief Model as an Explanatory Framework in Communication Research: Exploring Parallel, Serial, and Moderated Mediation. Health Commun. 2015;30(6):566-76.

45. Hansen EC. Successful Qualitative Health Research: A Practical Introduction: Maidenhead: Open University Press; 2006.

46. O'Cathain A, Murphy E, Nicholl J. Three techniques for integrating data in mixed methods studies. Bmj. 2010;341:c4587.

47. Vijayananthan A, Nawawi O. The importance of Good Clinical Practice guidelines and its role in clinical trials. Biomed Imaging Interv J. 2008;4(1).

For peer review only

For peer review only - http://bmjopen.bmj.com/site/about/guidelines.xhtml

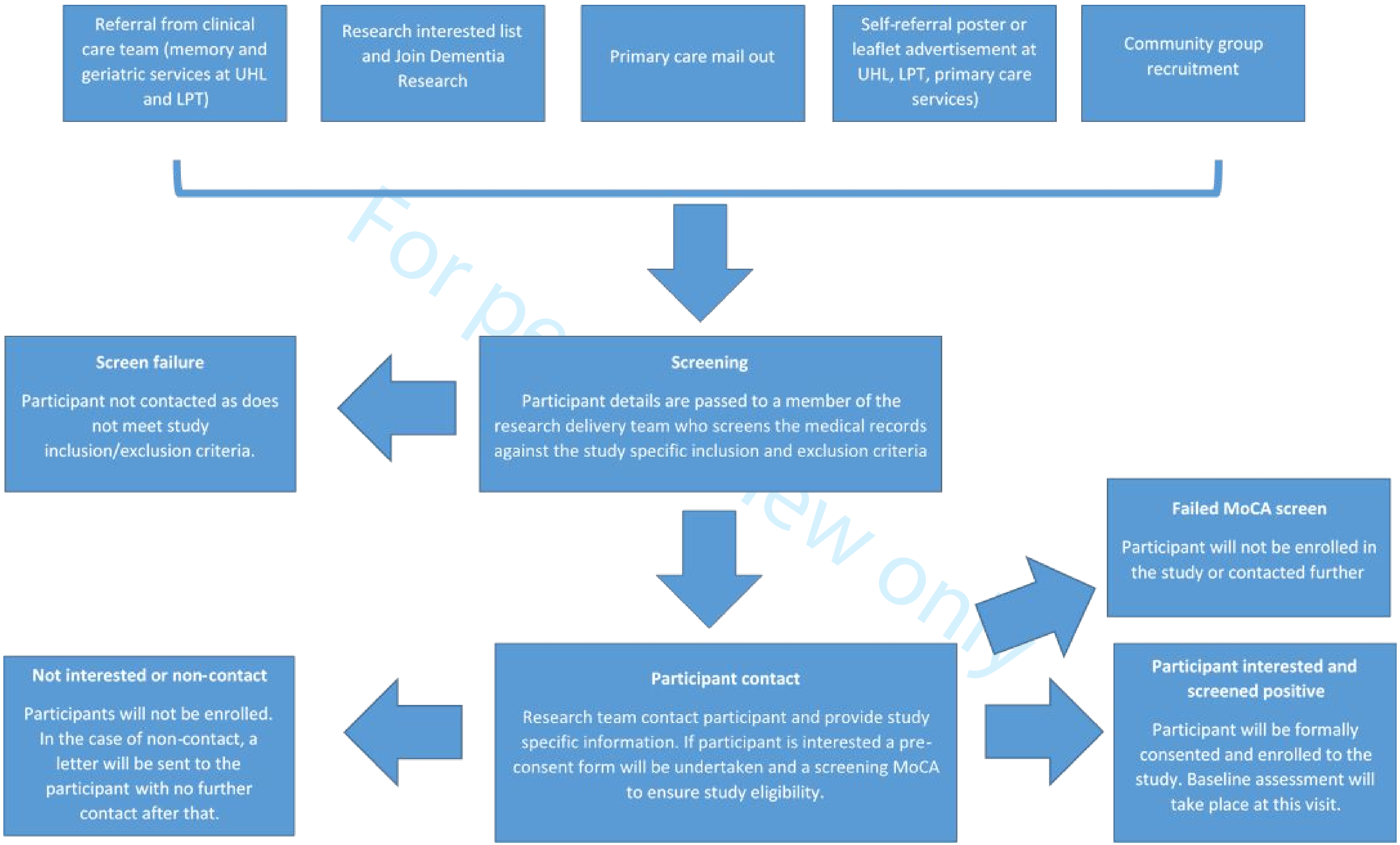

Figure 1. Flow chart of study recruitment procedures.

For peer review only - http://bmjopen.bmj.com/site/about/guidelines.xhtml

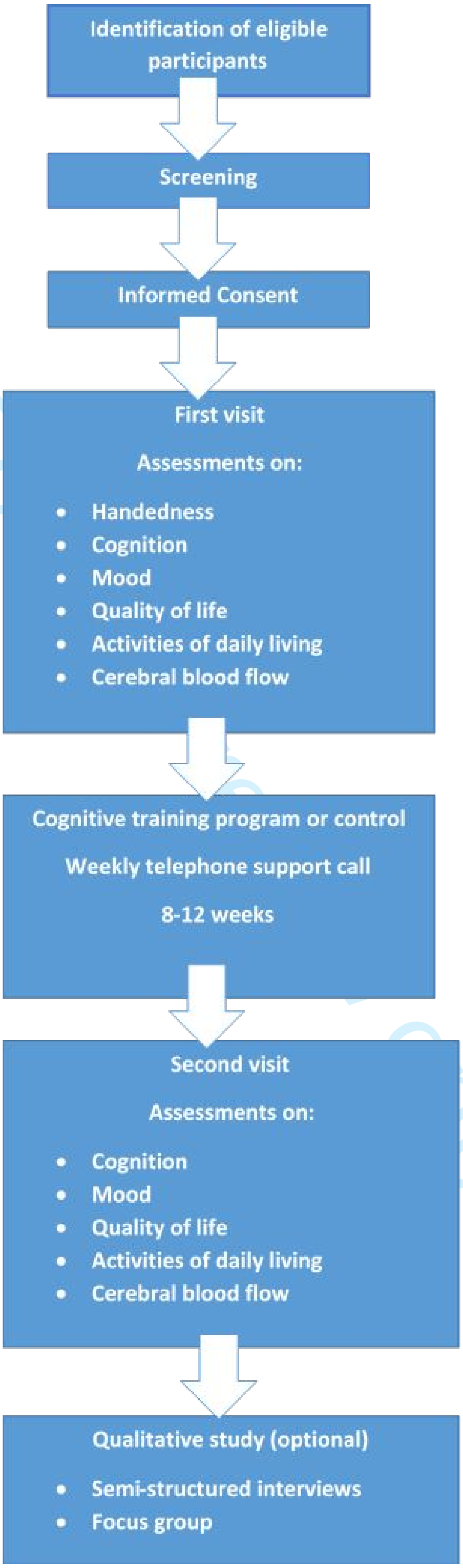

Figure 2. Flow chart of participant procedures during the study.

For peer review only - http://bmjopen.bmj.com/site/about/guidelines.xhtml

Supplementary information. Interview and focus group schedule of questions.

Research Questions

1. What are the barriers to brain training in patients with dementia?
2. What are the facilitators (benefits) to brain training in patients with dementia?
3. Could brain training programs be adapted further to support the participation of patients with dementia?
4. Are there any additional benefits to brain training programs not measured by traditional methods as perceived by the patients and their carers?
5. To explore the lived experience of the patient and their carer and the impact brain training has on them and their life

For peer review only

Section 1. Evaluating the Brain Training Program/ Study Design

- How did participants/carers find the program? (opening question)
- What were the positive aspects to completing the program? (RQ2/4)
- What were the negative aspects to completing the program? (RQ1)
- Did any issues or difficulties arise during the program and how did you manage these? (RQ1/2)
- Do you have any suggestions to improve the program for future studies? (RQ 3)

Section 2. Feasibility (RQ3/4)

- Was it difficult to achieve the minimum number of sessions required to complete the program and if yes, why?
- Were the study assessments the right duration?
- Was the frequency of study visits acceptable?
- Would you make any changes to the study visits to make the experience better for people with dementia?

Section 3. Impact on patients and their carers

- How did the program affect your day to day life? (RQ5)
- Did the frequency or duration of the training interfere with your day to day life? (RQ4)
- How do you feel the brain training program affected your mood? (RQ4)
- How do you feel the brain training program affected your memory? (RQ4)
- How do you feel the brain training program affected your ability to carry out day to day activates? (RQ4)

Section 4. The health belief model

1) Risk susceptibility
    a. How likely do you think you are to develop dementia? (healthy volunteers, MCI)
    b. How do you think undertaking a brain training program affects your risk of developing dementia? (HV, MCI)
2) Risk severity

a. If you were to develop dementia, how serious do you think the consequences would be for you? (HV, MCI)
b. How severe do you think your memory difficulties are? (AzD, MCI)
c. How do you feel the brain training program has affected the severity of your symptoms? (AzD, MCI)

3) Benefits to action
   a. What helped you to complete the brain training program? (RQ2)
4) Barriers to action
   a. What stopped you with you completing the brain training program? (RQ1)
5) Self-efficacy
   a. If you experienced difficulties, how did you overcome these to complete the program? (RQ1/2)
6) Cues to action
   a. Did anything support or prompt you to complete the program? (RQ1/2)
   b. Did any thoughts, feelings or symptoms affect you completing the program? (RQ1/2)

For peer review only

Data Security and Storage

Source documents are original documents, data, and records from which participants' CRF data are obtained. These include, but are not limited to, hospital records (from which medical history and previous and concurrent medication may be summarised into the CRF), clinical and office charts, laboratory and pharmacy records, radiographs/scan reports, and correspondence, interview and focus group recordings and transcripts.

CRF entries will be considered source data if the CRF is the site of the original recording (e.g., there is no other written or electronic record of data). In this study the CRF will be used as the source document for; cognition (ACE-III), mood (GDS-15), function/ADLs (Lawton-IADL), quality of life (DEMQOL), handedness (Edinburgh Handedness Inventory), and assessments of neurovascular function (TCD), blood pressure, heart rate and $ETCO_2$.

All documents will be stored safely in confidential conditions. On all study-specific documents, other than the signed consent, the participant will be referred to by the study participant number/code, not by name. All parameters (signals) that are collected during the measurement will be saved using a coded filename. The name and other identifying detail will NOT be included in any study data electronic file. All study data will be entered into Microsoft Excel for Windows. All files will be encrypted and stored on a password secured computer/laptop, which will have restricted access to members who are authorised on the authorisation log. All paper documentation will be stored in a locked filing cabinet, in a locked office, on University of Leicester premises that require identification to enter the building. The participants will be identified by a study specific participant number and/or code in any database. The name and any other identifying detail will NOT be included in any study data electronic file.

For peer review only

Reporting checklist for protocol of a clinical trial.

Based on the SPIRIT guidelines.

Instructions to authors

Complete this checklist by entering the page numbers from your manuscript where readers will find each of the items listed below.

Your article may not currently address all the items on the checklist. Please modify your text to include the missing information. If you are certain that an item does not apply, please write "n/a" and provide a short explanation.

Upload your completed checklist as an extra file when you submit to a journal.

In your methods section, say that you used the SPIRIT reporting guidelines, and cite them as:

Chan A-W, Tetzlaff JM, Altman DG, Laupacis A, Gøtzsche PC, Krleža-Jerić K, Hróbjartsson A, Mann H, Dickersin K, Berlin J, Doré C, Parulekar W, Summerskill W, Groves T, Schulz K, Sox H, Rockhold FW, Rennie D, Moher D. SPIRIT 2013 Statement: Defining standard protocol items for clinical trials. Ann Intern Med. 2013;158(3):200-207

		Reporting Item	Page Number
Title	#1	Descriptive title identifying the study design, population, interventions, and, if applicable, trial acronym	1
Trial registration	#2a	Trial identifier and registry name. If not yet registered, name of intended registry	4,15
Trial registration: data set	#2b	All items from the World Health Organization Trial Registration Data Set	n/a
Protocol version	#3	Date and version identifier	2
Funding	#4	Sources and types of financial, material, and other support	17
Roles and responsibilities: contributorship	#5a	Names, affiliations, and roles of protocol contributors	16
Roles and responsibilities:	#5b	Name and contact information for the trial sponsor	2

For peer review only

sponsor contact information			
Roles and responsibilities: sponsor and funder	#5c	Role of study sponsor and funders, if any, in study design; collection, management, analysis, and interpretation of data; writing of the report; and the decision to submit the report for publication, including whether they will have ultimate authority over any of these activities	17
Roles and responsibilities: committees	#5d	Composition, roles, and responsibilities of the coordinating centre, steering committee, endpoint adjudication committee, data management team, and other individuals or groups overseeing the trial, if applicable (see Item 21a for data monitoring committee)	16
Background and rationale	#6a	Description of research question and justification for undertaking the trial, including summary of relevant studies (published and unpublished) examining benefits and harms for each intervention	5
Background and rationale: choice of comparators	#6b	Explanation for choice of comparators	5,6
Objectives	#7	Specific objectives or hypotheses	7
Trial design	#8	Description of trial design including type of trial (eg, parallel group, crossover, factorial, single group), allocation ratio, and framework (eg, superiority, equivalence, non-inferiority, exploratory)	5
Study setting	#9	Description of study settings (eg, community clinic, academic hospital) and list of countries where data will be collected. Reference to where list of study sites can be obtained	8
Eligibility criteria	#10	Inclusion and exclusion criteria for participants. If applicable, eligibility criteria for study centres and individuals who will perform the interventions (eg, surgeons, psychotherapists)	9
Interventions: description	#11a	Interventions for each group with sufficient detail to allow replication, including how and when they will be administered	10
Interventions: modifications	#11b	Criteria for discontinuing or modifying allocated interventions for a given trial participant (eg, drug dose change in response to harms, participant request, or improving / worsening disease)	12
Interventions: adherance	#11c	Strategies to improve adherence to intervention protocols, and any procedures for monitoring adherence (eg, drug tablet return;	10,11

For peer review only

		laboratory tests)	
Interventions: concomitant care	#11d	Relevant concomitant care and interventions that are permitted or prohibited during the trial	9
Outcomes	#12	Primary, secondary, and other outcomes, including the specific measurement variable (eg, systolic blood pressure), analysis metric (eg, change from baseline, final value, time to event), method of aggregation (eg, median, proportion), and time point for each outcome. Explanation of the clinical relevance of chosen efficacy and harm outcomes is strongly recommended	11,12
Participant timeline	#13	Time schedule of enrolment, interventions (including any run-ins and washouts), assessments, and visits for participants. A schematic diagram is highly recommended (see Figure)	11
Sample size	#14	Estimated number of participants needed to achieve study objectives and how it was determined, including clinical and statistical assumptions supporting any sample size calculations	13,14
Recruitment	#15	Strategies for achieving adequate participant enrolment to reach target sample size	8
Allocation: sequence generation	#16a	Method of generating the allocation sequence (eg, computer-generated random numbers), and list of any factors for stratification. To reduce predictability of a random sequence, details of any planned restriction (eg, blocking) should be provided in a separate document that is unavailable to those who enrol participants or assign interventions	10,11
Allocation concealment mechanism	#16b	Mechanism of implementing the allocation sequence (eg, central telephone; sequentially numbered, opaque, sealed envelopes), describing any steps to conceal the sequence until interventions are assigned	10,11
Allocation: implementation	#16c	Who will generate the allocation sequence, who will enrol participants, and who will assign participants to interventions	10,11
Blinding (masking)	#17a	Who will be blinded after assignment to interventions (eg, trial participants, care providers, outcome assessors, data analysts), and how	11
Blinding (masking): emergency	#17b	If blinded, circumstances under which unblinding is permissible, and procedure for revealing a participant's allocated intervention during	11

For peer review only

unblinding		the trial	
Data collection plan	#18a	Plans for assessment and collection of outcome, baseline, and other trial data, including any related processes to promote data quality (eg, duplicate measurements, training of assessors) and a description of study instruments (eg, questionnaires, laboratory tests) along with their reliability and validity, if known. Reference to where data collection forms can be found, if not in the protocol	11,12
Data collection plan: retention	#18b	Plans to promote participant retention and complete follow-up, including list of any outcome data to be collected for participants who discontinue or deviate from intervention protocols	10,11
Data management	#19	Plans for data entry, coding, security, and storage, including any related processes to promote data quality (eg, double data entry; range checks for data values). Reference to where details of data management procedures can be found, if not in the protocol	12
Statistics: outcomes	#20a	Statistical methods for analysing primary and secondary outcomes. Reference to where other details of the statistical analysis plan can be found, if not in the protocol	13,14
Statistics: additional analyses	#20b	Methods for any additional analyses (eg, subgroup and adjusted analyses)	14
Statistics: analysis population and missing data	#20c	Definition of analysis population relating to protocol non-adherence (eg, as randomised analysis), and any statistical methods to handle missing data (eg, multiple imputation)	13,14
Data monitoring: formal committee	#21a	Composition of data monitoring committee (DMC); summary of its role and reporting structure; statement of whether it is independent from the sponsor and competing interests; and reference to where further details about its charter can be found, if not in the protocol. Alternatively, an explanation of why a DMC is not needed	16
Data monitoring: interim analysis	#21b	Description of any interim analyses and stopping guidelines, including who will have access to these interim results and make the final decision to terminate the trial	16
Harms	#22	Plans for collecting, assessing, reporting, and managing solicited and spontaneously reported adverse events and other unintended effects of trial interventions or trial conduct	16
Auditing	#23	Frequency and procedures for auditing trial conduct, if any, and	n/a

For peer review only

		whether the process will be independent from investigators and the sponsor	
Research ethics approval	#24	Plans for seeking research ethics committee / institutional review board (REC / IRB) approval	16
Protocol amendments	#25	Plans for communicating important protocol modifications (eg, changes to eligibility criteria, outcomes, analyses) to relevant parties (eg, investigators, REC / IRBs, trial participants, trial registries, journals, regulators)	16
Consent or assent	#26a	Who will obtain informed consent or assent from potential trial participants or authorised surrogates, and how (see Item 32)	8
Consent or assent: ancillary studies	#26b	Additional consent provisions for collection and use of participant data and biological specimens in ancillary studies, if applicable	n/a
Confidentiality	#27	How personal information about potential and enrolled participants will be collected, shared, and maintained in order to protect confidentiality before, during, and after the trial	12
Declaration of interests	#28	Financial and other competing interests for principal investigators for the overall trial and each study site	16
Data access	#29	Statement of who will have access to the final trial dataset, and disclosure of contractual agreements that limit such access for investigators	16
Ancillary and post trial care	#30	Provisions, if any, for ancillary and post-trial care, and for compensation to those who suffer harm from trial participation	n/a
Dissemination policy: trial results	#31a	Plans for investigators and sponsor to communicate trial results to participants, healthcare professionals, the public, and other relevant groups (eg, via publication, reporting in results databases, or other data sharing arrangements), including any publication restrictions	15
Dissemination policy: authorship	#31b	Authorship eligibility guidelines and any intended use of professional writers	n/a
Dissemination policy: reproducible research	#31c	Plans, if any, for granting public access to the full protocol, participant-level dataset, and statistical code	n/a
Informed consent materials	#32	Model consent form and other related documentation given to participants and authorised surrogates	n/a

For peer review only

Biological specimens	#33	Plans for collection, laboratory evaluation, and storage of biological specimens for genetic or molecular analysis in the current trial and for future use in ancillary studies, if applicable	n/a

The SPIRIT checklist is distributed under the terms of the Creative Commons Attribution License CC-BY-ND 3.0. This checklist was completed on 08. November 2018 using http://www.goodreports.org/, a tool made by the EQUATOR Network in collaboration with Penelope.ai

For peer review only

BMJ Open

The Effects of Brain Training on Brain Blood Flow (The Cognition and Flow Study -CogFlowS): A Protocol for A Feasibility Randomised Controlled Trial of Cognitive Training in Dementia.

Journal:	*BMJ Open*
Manuscript ID	bmjopen-2018-027817.R1
Article Type:	Protocol
Date Submitted by the Author:	07-Mar-2019
Complete List of Authors:	Beishon, Lucy; University of Leicester College of Medicine Biological Sciences and Psychology,<br>Evley, Rachel<br>Panerai, Ronney<br>Subramaniam, Hari<br>Mukaetova-Ladinska, Elizabeta<br>Robinson, Thompson; University of Leicester College of Medicine Biological Sciences and Psychology, Cardiovascular Sciences<br>Haunton, Victoria
**Primary Subject Heading**:	Mental health
Secondary Subject Heading:	Cardiovascular medicine
Keywords:	Neuroimaging, Cerebral haemodynamics, Alzheimer's disease, Mild cognitive impairment, Transcranial Doppler ultrasonography, cognitive impairment

SCHOLARONE™
Manuscripts

Title

The Effects of Brain Training on Brain Blood Flow (The Cognition and Flow Study -CogFlowS): A Protocol for A Feasibility Randomised Controlled Trial of Cognitive Training in Dementia.

Authors

Lucy Beishon[a], Rachel Evley[b] Ronney B Panerai[a,c], , Hari Subramaniam[d], Elizabeta Mukaetova-Ladinska[d,e], Thompson G Robinson[a,b], Victoria J Haunton[a,b].

Affiliations

[a] University of Leicester, Department of Cardiovascular Sciences, Leicester, UK

[b] Division of Clinical Neuroscience, University of Nottingham, UK

[c] NIHR Leicester Biomedical Research Centre, British Heart Foundation Cardiovascular Research Centre, Glenfield Hospital, Leicester, UK

[d] The Evington Centre, Leicestershire Partnership NHS Trust, Leicester, UK

[e] University of Leicester, Department of Neuroscience, Psychology and Behaviour, Leicester, UK

Corresponding Author

Lucy Beishon

Lb330@le.ac.uk

Room 228, Level 2 Clinical Sciences Building

Leicester Royal Infirmary

Leicester

LE2 7LX

For peer review only - http://bmjopen.bmj.com/site/about/guidelines.xhtml

Tel: 0116 252 3134

Key words

Cognitive impairment

Neuroimaging

Cerebral haemodynamics

Alzheimer's disease

Mild cognitive impairment

Transcranial Doppler ultrasonography

Word count

3679

Protocol date and version number

30/10/2018, version 2

Trial sponsor

Dr Michelle Meussel

Research Governance Office, Leicester General Hospital

Leicester

LE5 4PW

01162584077

uolsponsor@le.ac.uk

For peer review only

Abstract

Introduction

Cognitive training is an emerging non-pharmacological treatment to improve cognitive and physical function in mild cognitive impairment (MCI) and early Alzheimer's disease (AD). Abnormal brain blood flow is a key process in the development of cognitive decline. However, no studies have explored the effects of cognitive training on brain blood flow in dementia. The primary aim of this study is to assess the feasibility for a large scale, randomised controlled trial of cognitive training in healthy older adults (HC), MCI, and early AD.

Methods and analysis

This study will recruit 60 participants, in three sub-groups of twenty (MCI, HC, AD), from primary, secondary, and community services. Participants will be randomised to an eight to twelve week computerised cognitive training programme (five x 30 minute sessions per week), or waiting-list control. Participants will undergo baseline and follow-up assessments of: mood, cognition, quality of life, and activities of daily living. Cerebral blood flow will be measured at rest and during task activation (pre- and post-training) by bilateral transcranial Doppler ultrasonography, alongside heart rate (3-lead ECG), end-tidal CO_2 (capnography), and beat-to-beat blood pressure (Finometer). Participants will be offered to join a focus group or semi-structured interview to explore barriers and facilitators to cognitive training in patients with dementia. Qualitative data will be analysed using framework analysis, and data will be integrated using mixed methods matrices.

Ethics and dissemination

Bradford Leeds Research Ethics committee awarded a favorable opinion (18/YH/0396). Results of the study will be published in peer reviewed journals, and presented at national and international conferences on ageing and dementia.

For peer review only

Registration

ClinicalTrials.Gov unique identifier: NCT03656107.

Article summary: Strengths and limitations (5 bullet points)

- This study uses a mixed methods approach to investigate the feasibility of cognitive training in healthy older adults, mild cognitive impairment, and Alzheimer's disease
- This study includes an assessment of the effects of cognitive training on neurovascular function, by transcranial Doppler ultrasonography
- This study includes a broad range of outcome measures to take a holistic approach to evaluating cognitive training in dementia
- The primary aim of this study is to identify the feasibility of this protocol in patients with cognitive impairment, and as such is not a fully powered trial
- This study is limited to identifying these outcomes in healthy older adults, mild cognitive impairment, and Alzheimer's disease with mild to moderate deficits

For peer review only

Introduction

The incidence of dementia is rising as the population ages, with 46.8 million people living with dementia worldwide (1, 2). By 2030, this is projected to rise to 75 million (1, 2). However, there are currently few diagnostic or therapeutic strategies available to offer these patients. Dementia is a progressive condition characterised by gradual loss of cognitive and/or non-cognitive higher functions (i.e. language, visuospatial, attention) (3). Mild cognitive impairment (MCI) is characterised by subjective and objective decline in cognitive function, but with preserved functional independence in daily living (4). It has become increasingly recognised that deranged vascular function is an early contributor to the deposition of amyloid plaques and tau tangles in Alzheimer's disease (AD), and that these pathologies exacerbate one another (two-hit hypothesis) (5). In a recent systematic review and meta-analysis, we demonstrated clear abnormalities in vascular function across a number of imaging modalities at the MCI stage (6). Thus, treatments that can improve cerebral perfusion or vascular function could represent an early treatment option for dementia (6). In this study, the target populations are: healthy older adults (as controls), adults with AD, and MCI, to capture the effects of cognitive training on vascular physiology at an early stage in the dementia process.

The Cognition and Flow Study (CogFlowS) is a feasibility, parallel, randomised controlled trial (RCT) to examine the use of a cognitive training programme in adults with MCI, early AD and healthy older adults (HC). In addition to measuring cognitive and functional outcomes, this study will also use transcranial Doppler ultrasonography (TCD) to measure changes in cerebral blood flow (CBF) before and after a 12-week cognitive training program.

Cognitive interventions can be considered as three broad categories: cognitive training, cognitive rehabilitation and cognitive stimulation (7, 8). Cognitive training describes a structured, guided program of standardised tasks designed to provide practice or training within a specified cognitive domain(s), to translate into functional benefits (7, 8). This is distinguished from cognitive rehabilitation, where patient centered approaches and goals are used to improve everyday function,

For peer review only - http://bmjopen.bmj.com/site/about/guidelines.xhtml

rather than improve cognition function (7-9), and cognitive stimulation which aims to generally improve cognition, social function and quality of life (10). Cognitive training is attractive in that it offers a cost effective, non-invasive and acceptable intervention to patients, with no reported adverse effects (11). However, high quality RCT evidence is lacking in studies of cognitive training in dementia (8, 9, 12, 13). Two previous Cochrane reviews (8, 9), highlighted that the evidence was sparse, with many of the studies under-powered, of low-to-moderate quality, and with relatively few for MCI (8, 9). Recent systematic reviews of cognitive training in MCI have demonstrated moderate benefits in verbal learning and memory, global cognition, non-verbal learning, working memory, attention and psychosocial functioning (12, 14). Thus far, studies of cognitive training in dementia have been heterogeneous in study design, participants included, type and definition of training, outcome measures, and duration and intensity of intervention (8, 9, 13, 15, 16). This hampers the adequate meta-analysis of data, and of conclusions that can be drawn from pooled analyses (13, 17). Few studies have included neuroimaging outcomes to explore brain plasticity or neural mechanisms underlying changes in cognition after cognitive training (17). Therefore, the CogFlowS study not only addresses the development of a future high quality RCT of cognitive training in dementia, but also aims to fill the evidence gap on the use of TCD to assess neurovascular plasticity.

TCD is a non-invasive, ultrasound based imaging modality that allows measurements of CBF velocity (CBFv) at rest (18) and following activation by cognitive tasks (19). TCD has a number of advantages over other functional imaging techniques, such as: magnetic resonance imaging (MRI) and positron emission tomography (PET), including lower cost, portability, relative ease of operator training, high temporal resolution and higher acceptability to patients (20, 21). TCD is particularly suited to older people, and those with dementia, as it can be used in patients with pacemakers, metal implants and claustrophobia (20). The use of TCD is a novel method to investigate neurovascular training effects and for markers predictive of which patients may benefit from particular cognitive training programmes. This would allow the development of a personalised approach to developing cognitive training programmes for people with dementia.

For peer review only - http://bmjopen.bmj.com/site/about/guidelines.xhtml

For peer review only

The use of neuroimaging outcomes has also been limited in cognitive training studies (17, 22), but understanding the training effects occurring at the physiological level may provide important mechanistic information to any potential benefits conferred by cognitive training (23-25). Belleville et al demonstrated increased parietal activation in patients with MCI, following a multi-modal cognitive training program designed to improve episodic memory (23). In a near-infrared spectroscopy study by Vermeij et al, patients with MCI had greater training benefits at high load working memory where participants had greater pre-frontal activation (26), whereas baseline global and hippocampal atrophy were predictive of poorer training gains in healthy older adults and those with MCI (24). Therefore, imaging biomarkers can potentially be used to predict and tailor interventions to individual patient's needs (24, 25). Whilst a number of studies have demonstrated positive structural and functional effects of cognitive training (25, 27, 28), they have largely focused on healthy older adults (27), with the use of structural (27) or functional MRI (23, 24) and PET (29, 30); there have been no TCD studies examining changes in neurovascular response to cognitive training in patients with cognitive impairment. In addition to the inclusion of neuroimaging, this study utilises a mixed methods approach. This study includes a qualitative component to explore any benefits, problems or barriers to engaging with the training program, and to identify any benefits not measured by traditional outcomes.

The primary objective for this study is to determine the feasibility for a large scale RCT of cognitive training in HC, AD, and MCI. Secondary objectives seek to identify any clinical benefits of a cognitive training program in terms of: activities of daily living, cognition, mood, and quality of life, and TCD-measured task activation responses in brain blood flow. In addition, the qualitative study seeks to explore barriers and facilitators to the cognitive training programme, how programs can be adapted to support participation for patients living with dementia, and any additional benefits not measured through traditional methods as perceived by patients and their carers. Finally, we plan to explore the lived experience of the patient and their carer, and the impact cognitive training has on them and their life.

For peer review only - http://bmjopen.bmj.com/site/about/guidelines.xhtml

Methods and analysis

This protocol was reported in accordance with the SPIRIT checklist (31), which is included in Supplementary information.

Patient and public involvement

During the design phase of this study, a patient and public involvement (PPI) group was consulted, comprised of research network volunteers from the Alzheimer's society who were either people living with cognitive impairment or their carers. The group reviewed the study design, research questions and outcome measures, and a number of changes were made as a result. These included: providing an option of a focus group or interview to participants, changes to the recruitment procedures, and the design of the cognitive training programme. All study participants will receive a lay summary at the end of the study explaining what the outcomes of the study were. A further PPI meeting was held following successful funding to refine the study documentation and procedures for participants. The trial will be overseen by a trial steering committee (TSC), consisting of patient and public members, and independent academics. The TSC will review the trial conduct on a six monthly basis. As this is a feasibility trial, the burden of intervention is to be assessed in terms of its acceptability to patients and their carers.

Sample selection

HC will be recruited through poster advertisement, friends, family, and carers of enrolled patient participants, Join Dementia Research, and the research interested list at the University of Leicester. Participants with a diagnosis of MCI, or AD, will be recruited through poster advertisement, secondary care services (outpatient clinics within the University Hospitals of Leicester (acute) and the Leicestershire Partnership (mental health) NHS Trusts), research interested lists, Join Dementia Research, and local community groups. In addition, participants will be sought through letter

For peer review only

For peer review only - http://bmjopen.bmj.com/site/about/guidelines.xhtml

invitation from their GP surgery. Participants will be allowed a minimum of 24 hours to decide if they would like to enrol in the study, and to contact the researcher or research delivery team at the Leicestershire Partnership Trust, who will arrange for them to undergo an eligibility assessment, formal consent, and study enrolment. As part of the eligibility assessment participants will undergo a screening cognitive assessment (Montreal Cognitive Assessment – MoCA) to ensure deficits are classified as mild to moderate. Participants will be consented for this, and the MoCA will not form part of the baseline assessments. For participants who lack capacity to consent to the study, a personal consultee declaration will be completed by a friend, relative, or carer, in addition to verbal assent from the participant themselves where possible. Flow charts of the recruitment process and participant procedures can be seen in Figures 1 and 2, respectively.

The planned recruitment target for this study is 60 participants, divided into three sub-groups of twenty (HC, AD, and MCI). As the primary aim of this study is feasibility, a formal sample size calculation has not been performed. Recruiting between 24 and 50 participants for a feasibility study is acceptable and therefore the planned target of 60 participants is sufficiently large enough whilst giving a margin for drop-outs and loss to follow-up (32, 33).

The following inclusion and exclusion criteria were designed to identify a sample of participants that will be able to engage with a cognitive training programme, and to exclude any major medical co-morbidities that can affect CBF:

Inclusion criteria

1) Healthy controls will be free of any medical co-morbidity or medication that could adversely affect cognition. Volunteers with well-controlled co-morbidities (i.e. hypertension, diabetes, will be considered for inclusion)

2) MCI as defined by NIA/AA 2011 (34) and Petersen criteria (4)

For peer review only - http://bmjopen.bmj.com/site/about/guidelines.xhtml

For peer review only

3) AD as defined by the NIA/AA 2011 criteria (34)

And:

4) Deficits will be mild to moderate as defined by Montreal Cognitive Assessment (MoCA) score of >9 for participants with MCI and AD (35-37).

5) Willing to participate

6) Capacity to consent to the study/personal consultee

7) Patients on and off anti-dementia medications will be included (acetylcholinesterase inhibitors, glutamate receptor antagonists)

8) Good understanding of written and spoken English

9) Age >50 years

10) Access to the internet and a computer/laptop device.

Exclusion criteria

1) Healthy controls with any medical co-morbidity or medication that could adversely affect cognition, or poorly controlled medical co-morbidities (i.e. hypertension, diabetes)

2) Unwilling to take part

3) Unable to consent/no personal consultee

4) Major medical co-morbidity: severe heart failure (ejection fraction <20%), carotid artery stenosis, severe respiratory disease, major stroke

5) Pregnancy, planning pregnancy, or lactating

6) Inadequate bilateral TCD windows

7) Participants already enrolled into other interventional studies that would confound study results

8) Insufficient understanding of written and spoken English

For peer review only - http://bmjopen.bmj.com/site/about/guidelines.xhtml

9) Age <50 years

10) No access to the internet and a computer/laptop device

Intervention

Participants randomised to the intervention will complete a twelve week cognitive training programme. Participants will be required to complete five, 30 minute sessions per week for twelve weeks. The cognitive training programme will be provided by Lumosity© as part of a collaboration through the Human Cognition Project. Lumosity© is commercially available software, developed by a group of neuropsychologists, which has been used across several studies of brain training and disciplines (38-40). These studies have been included in a number of systematic reviews and meta-analysis (12, 41). It is a multi-domain, online-based cognitive training tool, which is relatively easy to use and administer. It has been designed to adapt to the individual's cognitive function in order to administer the training program to their individual level of cognitive function. Exercises will be selected from Lumosity's© commercially available online software which have been pre-classified to target the following cognitive domains: attention, memory, visuospatial, verbal fluency, and language. These five cognitive domains will be evaluated through formal cognitive testing, and through the neurovascular coupling assessment. Compliance will be monitored through Lumosity© online software which will log and track the number of minutes and times per week a participant has spent training, in order to calculate the dose of training to which each participant has been exposed.

Randomisation

Randomisation will be performed using Sealed Envelope©, by the researcher. This is an online-based randomisation tool which uses random permuted blocks to allocate participants to waiting list control or intervention. Participants will be enrolled and assigned a PIN consecutively, and randomised to a treatment arm corresponding to the PIN. Given the nature of the trial, it is not possible to blind participants to the intervention. The Investigator will be providing weekly telephone support for the intervention group, in addition to undertaking baseline and follow-up measurements, and therefore

For peer review only - http://bmjopen.bmj.com/site/about/guidelines.xhtml

blinding of the investigator is also not possible. However, data analysis will be blinded by generating a batch-anonymised data set. Randomisation will be undertaken at the initial visit. Once participants have been randomised to the intervention arm, they will be provided with information on how to access and use the cognitive training programme at home.

Data collection and integration

All participants (intervention and control groups) will be required to attend two assessments: baseline, and follow-up at eight to twelve weeks post-training. Assessments will be carried out at the participant's home, or designated research space at the Leicestershire Partnership Trust, or Cerebral Haemodynamics in Ageing and Stroke Medicine (CHiASM) research space. Data will be collected on demographics, medical history, and medication use. Assessments of: cognition [Addenbrooke's cognitive examination III (ACE-III)], mood (Geriatric Depression Scale 15), activities of daily living (Lawton instrumental activities of daily living), and quality of life (Dementia Rated Quality of Life), will be carried out at both visits. An assessment of neurovascular function will be carried out at the CHiASM research space, a temperature controlled room, free of noise and distraction. Participants will be seated throughout the protocol. All measurements will be made at rest (five minute baseline recording), and during cerebral activation (selected cognitive tasks). CBFv will be measured continuously by insonation of the bilateral middle cerebral arteries (MCA) (DWL Doppler Box), using 2 MHz probes, fixed in place with a headframe. Beat-to-beat blood pressure will be measured continuously using a Finometer cuff on the non-dominant middle finger (Finapres Medical Systems, Amsterdam), and calibrated using a brachial blood pressure recording (UA767 BP monitor). End-tidal CO_2 will be measured by capnography using nasal cannulae (Capnochek Plus), and the R-R interval will be recorded using a 3-lead ECG. The task activation protocol has been published previously (42, 43). In brief, participants will be presented with five tasks selected from the ACE-III, and percentage increase in CBFv from resting baseline will be calculated at task initiation. The tasks will be selected

For peer review only - http://bmjopen.bmj.com/site/about/guidelines.xhtml

for five cognitive domains: attention, memory, visuospatial, language, and verbal fluency, based on recent studies conducted by this group (43, 44).

At least one week after completion of the follow-up assessment, participants with a diagnosis of MCI, or AD, will be invited to attend a semi-structured interview or focus group. The schedule of questions can be seen in supplementary information. The schedule of questions has been framed around the six constructs of the health belief model (risk susceptibility, risk severity, benefits to action, barriers to action, self-efficacy, and cues to action) (45) to evaluate the cognitive training programme, and the experience of the participant and their carer. Interviews and focus groups will be recorded continuously with a digital audio recorder, and notes of non-verbal and paralinguistic clues will be made. All digital recordings will be transcribed within five to seven days of the interview or focus group. Semi-structured interviews are an iterative process, following the first couple of interviews the transcripts will be analysed and the themes and concepts emerging from these initial data will be further explored at the following interviews.

Data security and storage will be conducted in line with general data protection regulations (https://eugdpr.org/), and details regarding data security and storage can be seen in Supplementary information.

Trial discontinuation

This is not a drug intervention trial, and therefore interim analysis will not be required. Criteria for trial discontinuation are as follows: ineligibility, significant protocol deviation, significant non-adherence to the programme, an adverse event or disease progression resulting in inability to comply with study procedures, consent withdrawn, or lost to follow-up.

Data analysis and interpretation

For peer review only

The difference for each participant between baseline and follow-up assessment for each of the continuous outcome measures described above will be reported along with the overall mean difference.

CBFv data will be recorded and stored in the PHYSIDAS system, and analysed offline. Data will be visually inspected for quality, and files rejected where quality is deemed to be poor, with reasons. Peak percentage change in CBFv will be calculated relative to a twenty second baseline, prior to task initiation.

The findings from the semi-structured interviews and focus groups will be evaluated using framework analysis (41). The digital recordings of the interviews and focus groups will be transcribed verbatim, the transcripts will be read in detail, i.e. line-by-line, and open codes will be formed categorising and conceptualising the responses and identifying the major themes. Two researchers LB & RE will independently code the first few transcripts to ensure consistency in coding.

Following this initial coding the analytical framework will be developed, this is an iterative process and will develop through coding of additional transcripts. Once the final transcript has been coded the analytic framework will be used to generate the framework matrix. The framework matrix will be developed in NVivo 11 (QSR International), and allow for the recognition of patterns and outliers within the data. Respondent validation will be used to establish a degree of correspondence between the researcher's views and those of the research participant (46).

Data from the quantitative and qualitative arms of the study will be integrated using mixed methods approaches of triangulation and mixed methods matrices (47).

Statistical analysis and sample size calculation

Data will be checked for normality, and non-normally distributed data will be appropriately transformed, and parametric tests applied. Data will be reported as mean (standard deviation) for continuous variables, and number (percentage) for nominal data. Differences in baseline

For peer review only - http://bmjopen.bmj.com/site/about/guidelines.xhtml

demographics between the intervention and control groups will be analysed within population group (HC/AD/MCI) using Chi-Square for nominal data, and independent t-testing for continuous data. Any baseline differences in demographics between population groups will also be reported. To assess the impact of the intervention and population group on each of these differences, a two-way ANOVA will be carried out. The interaction between population group and treatment group will be analysed to assess if the difference between the treatment groups is different between the population groups. Post-hoc testing will be conducted by Tukey to analyse for main effects. Data will be recorded in Microsoft Excel. Statistical analyses will be performed using the latest version of SPSS for Windows (SPSS v.24), and graphs will be produced using the latest version of GraphPad Prism for Windows.

Study limitations

This is a feasibility study, and thus will not be powered adequately to detect changes in the secondary end points outlined above. However, given the novel aspects of this study protocol, such as the inclusion of a TCD-measured changes in CBF responses, and the mixed methods design, this requires evaluation of the feasibility before moving to a larger, fully powered trial of cognitive training in dementia. Furthermore, this study will include assessments of patient experience and tolerability to facilitate this. Although TCD affords excellent temporal resolution in the measurement of CBFv, the MCA supplies blood to approximately 80% of the cerebral cortex, and therefore reflects a global measure of perfusion, and cannot discriminate where changes are occurring, due to poor spatial resolution. Techniques such as MRI, and PET scans afford better spatial resolution, but have their own limitations, particularly in older patients with cognitive deficits. TCD-measured CBFv relies on the assumption that the vessel diameter remains constant, despite fluctuations in CO_2 and blood pressure (18). This study elected to focus on healthy older adults, MCI and AD in the first instance. Deficits in CBFv have been demonstrated in both MCI and AD, but inclusion of participants with vascular cognitive impairment would provide further information on the capacity for plasticity to neurovascular physiology amongst dementia sub-types. In addition, only participants with mild to moderate deficits

For peer review only - http://bmjopen.bmj.com/site/about/guidelines.xhtml

will be included, given that data thus far do not support the use of cognitive training in more advanced dementia (9, 12). Only participants who are undertaking the training programme will be offered telephone support, as it is primarily to support technical issues with delivery of the programme. However, this could introduce a placebo effect, and future studies would ideally correct for this by providing support to both groups. Finally, one investigator is undertaking both baseline and follow-up assessments, and providing telephone support, which carries a risk of introducing researcher-bias into the study. As this is primarily a feasibility study, this is of lesser importance here, but a future trial should ideally have separate team members conducting these different roles to minimise the risk of bias.

Ethics and dissemination

Ethics and safety considerations

This study has been reviewed by the Bradford Leeds Research Ethics Committee (REC), and was granted a favorable opinion on the 7/11/18 (18/YH/0396). All participants with capacity in this study will provide informed, written consent, and the study will be conducted in accordance with the Declaration of Helsinki and Good Medical Practice guidelines (48). Given the nature of dementia, not all participants will have capacity to consent to the study. In this instance, a personal consultee (friend, relative, or carer) will be consulted for their opinion on whether the participant is likely to have wanted to participate in this study, and they will be asked to sign a consultee declaration form. It was not considered ethical to exclude participants on the grounds of capacity, given the nature of the disease studied.

Dissemination

The results from this study will be submitted to peer-reviewed journals in ageing and dementia research, and presented at national and international conferences on these themes. We anticipate the results will be presented and published in late 2020, early 2021. In addition, the results will be

For peer review only - http://bmjopen.bmj.com/site/about/guidelines.xhtml

disseminated to members of the community through the Alzheimer's Society, community groups, podcasts, and social media.

Trial registration

This study has been registered on ClinicalTrials.gov (ref: NCT03656107). The trial was registered prior to initiation, and will be completed within six months of recruitment of the first study participant.

Ethics approval

Ethical approval was obtained from the Bradford Leeds Research Ethics Committee (18/YH/0396)

Competing interests

None.

Author Contributions

The quantitative arm of the study was conceived and designed by LB, VJH, RBP, and TGR. LB and RE designed the qualitative arm of the study. HS and EML reviewed and edited the protocol. All authors assisted in drafting this manuscript.

Trial committees and monitoring

The study will be monitored at six-monthly intervals throughout its duration by a trial steering committee, independent of the funder, consisting of lay and independent academic members. Each site will have a lead investigator who is responsible for the identification and recruitment of participants, data collection and completion for CRFs, follow-up measurements, and adherence to study protocols. Serious adverse events (SAEs) will be reported to the sponsor within one working day of identification, and the Sponsor will report all serious unexpected or adverse reactions to the ethics committee concerned. The chief investigator will submit an annual report to the ethics committee which lists all SAEs. Where necessary, protocol amendments will be submitted to the study sponsor or ethics committee for approval.

For peer review only - http://bmjopen.bmj.com/site/about/guidelines.xhtml

For peer review only

Provenance and peer review

Not commissioned.

Data sharing agreement

The data will be stored in the Leicester Research Archive after study completion, and data will be made available at request.

Funding

This study was reviewed and funded by the Dunhill Medical Trust, and LB is a Dunhill research training fellow (RTF1806\27). TGR is an NIHR Senior Investigator. This funding source had and will not have a role in the design, conduct, analyses, interpretation of the data, or decision to submit results. The cognitive training programme is being provided by Lumosity© free of charge, as part of an industry-institution collaboration through the Human Cognition Project. Lumosity© is providing advice and support on the composition and nature of the programme. However, this study is financially independent of Lumosity©, and Lumosity© cannot influence or change the results or outcomes of this study.

Acknowledgments

We would like to thank the members of the patient and public involvement group from the Alzheimer's Society for their contribution to the development of this protocol.

For peer review only

Figure Legends

Figure 1. Flow chart of study recruitment procedures.

Figure 2. Flow chart of participant procedures during the study.

For peer review only

For peer review only - http://bmjopen.bmj.com/site/about/guidelines.xhtml

References

1. Alzheimer's-society. Demography: Alzheimer's Society; 2016 [updated 2016-11-28. Available from: https://www.alzheimers.org.uk/info/20091/position_statements/93/demography.
2. Alzheimer's-Association. 2014 Alzheimer's disease facts and figures. Alzheimer's & dementia : the journal of the Alzheimer's Association. 2014;10(2):e47-92.
3. Robinson L, Tang E, Taylor JP. Dementia: timely diagnosis and early intervention. BMJ. 2015;350:h3029.
4. Petersen RC. Mild cognitive impairment as a diagnostic entity. J Intern Med. 2004;256(3):183-94.
5. Zlokovic BV. Neurovascular pathways to neurodegeneration in Alzheimer’s disease and other disorders. Nat Rev Neurosci. 2011;12(12):723-38.
6. Beishon L, Haunton VJ, Panerai RB, Robinson TG. Cerebral Hemodynamics in Mild Cognitive Impairment: A Systematic Review. J Alzheimers Dis. 2017;59(1):369-85.
7. Belleville S. Cognitive training for persons with mild cognitive impairment. Int Psychogeriatr. 2008;20(1):57-66.
8. Martin M, Clare L, Altgassen AM, Cameron MH, Zehnder F. Cognition-based interventions for healthy older people and people with mild cognitive impairment. The Cochrane database of systematic reviews. 2011(1):Cd006220.
9. Bahar-Fuchs A, Clare L, Woods B. Cognitive training and cognitive rehabilitation for persons with mild to moderate dementia of the Alzheimer's or vascular type: a review. Alzheimers Res Ther. 2013;5(4):35.
10. Woods B, Aguirre E, Spector AE, Orrell M. Cognitive stimulation to improve cognitive functioning in people with dementia. The Cochrane database of systematic reviews. 2012(2):Cd005562.
11. Gates NJ, Sachdev P. Is cognitive training an effective treatment for preclinical and early Alzheimer's disease? J Alzheimers Dis. 2014;42 Suppl 4:S551-9.
12. Hill NT, Mowszowski L, Naismith SL, Chadwick VL, Valenzuela M, Lampit A. Computerized Cognitive Training in Older Adults With Mild Cognitive Impairment or Dementia: A Systematic Review and Meta-Analysis. Am J Psychiatry. 2017;174(4):329-40.
13. Gates NJ, Sachdev PS, Fiatarone Singh MA, Valenzuela M. Cognitive and memory training in adults at risk of dementia: a systematic review. BMC Geriatr. 2011;11:55.
14. Li H, Li J, Li N, Li B, Wang P, Zhou T. Cognitive intervention for persons with mild cognitive impairment: A meta-analysis. Ageing Res Rev. 2011;10(2):285-96.
15. Huntley JD, Gould RL, Liu K, Smith M, Howard RJ. Do cognitive interventions improve general cognition in dementia? A meta-analysis and meta-regression. BMJ Open. 2015;5(4).
16. Reijnders J, van Heugten C, van Boxtel M. Cognitive interventions in healthy older adults and people with mild cognitive impairment: a systematic review. Ageing Res Rev. 2013;12(1):263-75.
17. Coyle H, Traynor V, Solowij N. Computerized and virtual reality cognitive training for individuals at high risk of cognitive decline: systematic review of the literature. Am J Geriatr Psychiatry. 2015;23(4):335-59.
18. Panerai RB. Transcranial Doppler for evaluation of cerebral autoregulation. Clin Auton Res. 2009;19(4):197-211.
19. Stroobant N, Vingerhoets G. Transcranial Doppler ultrasonography monitoring of cerebral hemodynamics during performance of cognitive tasks: a review. Neuropsychol Rev. 2000;10(4):213-31.
20. van Beek AH, Claassen JA, Rikkert MG, Jansen RW. Cerebral autoregulation: an overview of current concepts and methodology with special focus on the elderly. J Cereb Blood Flow Metab. 2008;28(6):1071-85.
21. Tomek A, Urbanova B, Hort J. Utility of transcranial ultrasound in predicting Alzheimer's disease risk. J Alzheimers Dis. 2014;42:S365-S74.

For peer review only - http://bmjopen.bmj.com/site/about/guidelines.xhtml

22. Vemuri P, Fields J, Peter J, Klöppel S. Cognitive interventions in Alzheimer's and Parkinson's diseases: emerging mechanisms and role of imaging. Curr Opin Neurol. 2016;29(4):405-11.
23. Belleville S, Clement F, Mellah S, Gilbert B, Fontaine F, Gauthier S. Training-related brain plasticity in subjects at risk of developing Alzheimer's disease. Brain. 2011;134(Pt 6):1623-34.
24. Vermeij A, Claassen JA, Dautzenberg PL, Kessels RP. Transfer and maintenance effects of online working-memory training in normal ageing and mild cognitive impairment. Neuropsychol Rehabil. 2016;26(5-6):783-809.
25. Belleville S, Bherer L. Biomarkers of Cognitive Training Effects in Aging. Curr Transl Geriatr Exp Gerontol Rep. 2012;1(2):104-10.
26. Vermeij A, Kessels RPC, Heskamp L, Simons EMF, Dautzenberg PLJ, Claassen JAHR. Prefrontal activation may predict working-memory training gain in normal aging and mild cognitive impairment. Brain Imaging and Behavior. 2016:1-14.
27. Engvig A, Fjell AM, Westlye LT, Moberget T, Sundseth O, Larsen VA, et al. Effects of memory training on cortical thickness in the elderly. Neuroimage. 2010;52(4):1667-76.
28. Yang H, Leaver AM, Siddarth P, Paholpak P, Ercoli L, St. Cyr NM, et al. Neurochemical and Neuroanatomical Plasticity Following Memory Training and Yoga Interventions in Older Adults with Mild Cognitive Impairment. Front Aging Neurosci. 2016;8.
29. Ciarmiello A, Gaeta MC, Benso F, Del Sette M. FDG-PET in the Evaluation of Brain Metabolic Changes Induced by Cognitive Stimulation in aMCI Subjects. Curr Radiopharm. 2015;8(1):69-75.
30. Forster S, Buschert VC, Teipel SJ, Friese U, Buchholz HG, Drzezga A, et al. Effects of a 6-month cognitive intervention on brain metabolism in patients with amnestic MCI and mild Alzheimer's disease. J Alzheimers Dis. 2011;26 Suppl 3:337-48.
31. Chan AW, Tetzlaff JM, Altman DG, Laupacis A, Gøtzsche PC, Krleža-Jerić K, et al. SPIRIT 2013 Statement: Defining Standard Protocol Items for Clinical Trials. Ann Intern Med. 2013;158(3):200-7.
32. Sim J, Lewis M. The size of a pilot study for a clinical trial should be calculated in relation to considerations of precision and efficiency. J Clin Epidemiol. 2012;65(3):301-8.
33. Julious SA. Sample sizes for clinical trials with normal data. Stat Med. 2004;23(12):1921-86.
34. Albert MS, DeKosky ST, Dickson D, Dubois B, Feldman HH, Fox NC, et al. The diagnosis of mild cognitive impairment due to Alzheimer's disease: recommendations from the National Institute on Aging-Alzheimer's Association workgroups on diagnostic guidelines for Alzheimer's disease. Alzheimer's & dementia : the journal of the Alzheimer's Association. 2011;7(3):270-9.
35. Saczynski JS, Inouye SK, Guess J, Jones RN, Fong TG, Nemeth E, et al. The Montreal Cognitive Assessment (MoCA): Creating a Crosswalk with the Mini-Mental State Examination. J Am Geriatr Soc. 2015;63(11):2370-4.
36. Borland E, Nagga K, Nilsson PM, Minthon L, Nilsson ED, Palmqvist S. The Montreal Cognitive Assessment: Normative Data from a Large Swedish Population-Based Cohort. J Alzheimers Dis. 2017;59(3):893-901.
37. Tan JP, Li N, Gao J, Wang LN, Zhao YM, Yu BC, et al. Optimal cutoff scores for dementia and mild cognitive impairment of the Montreal Cognitive Assessment among elderly and oldest-old Chinese population. J Alzheimers Dis. 2015;43(4):1403-12.
38. Kesler S, Hadi Hosseini SM, Heckler C, Janelsins M, Palesh O, Mustian K, et al. Cognitive training for improving executive function in chemotherapy-treated breast cancer survivors. Clin Breast Cancer. 2013;13(4):299-306.
39. Finn M, McDonald S. A single case study of computerised cognitive training for older persons with mild cognitive impairment. NeuroRehabilitation. 2014;35(2):261-70.
40. Ballesteros S, Prieto A, Mayas J, Toril P, Pita C, Ponce de León L, et al. Brain training with non-action video games enhances aspects of cognition in older adults: a randomized controlled trial. Front Aging Neurosci. 2014;6.
41. Lampit A, Hallock H, Valenzuela M. Computerized cognitive training in cognitively healthy older adults: a systematic review and meta-analysis of effect modifiers. PLoS Med. 2014;11(11):e1001756.

For peer review only - http://bmjopen.bmj.com/site/about/guidelines.xhtml

42. Beishon L, Williams CAL, Panerai RB, Robinson TG, Haunton VJ. Reproducibility of task activation using the Addenbrooke's cognitive examination in healthy controls: A functional Transcranial Doppler ultrasonography study. J Neurosci Methods. 2017.
43. Beishon LC, Williams CAL, Panerai RB, Robinson TG, Haunton VJ. The assessment of neurovascular coupling with the Addenbrooke's Cognitive Examination: a functional Transcranial Doppler Ultrasonographic Study. J Neurophysiol. 2017:jn.00698.2017.
44. Beishon L, Panerai RB, Robinson TG, Subramaniam H, Haunton VJ. The assessment of cerebrovascular response to a language task from the Addenbrooke's cognitive examination in cognitive impairment: A feasibility functional transcranial Doppler ultrasonography study. Journal of Alzheimer's Disease Reports. 2018;2:153-64.
45. Jones CL, Jensen JD, Scherr CL, Brown NR, Christy K, Weaver J. The Health Belief Model as an Explanatory Framework in Communication Research: Exploring Parallel, Serial, and Moderated Mediation. Health Commun. 2015;30(6):566-76.
46. Hansen EC. Successful Qualitative Health Research: A Practical Introduction: Maidenhead: Open University Press; 2006.
47. O'Cathain A, Murphy E, Nicholl J. Three techniques for integrating data in mixed methods studies. BMJ. 2010;341:c4587.
48. Vijayananthan A, Nawawi O. The importance of Good Clinical Practice guidelines and its role in clinical trials. Biomedical Imaging and Intervention Journal. 2008;4(1).

For peer review only

For peer review only - http://bmjopen.bmj.com/site/about/guidelines.xhtml

For peer review only

For peer review only - http://bmjopen.bmj.com/site/about/guidelines.xhtml

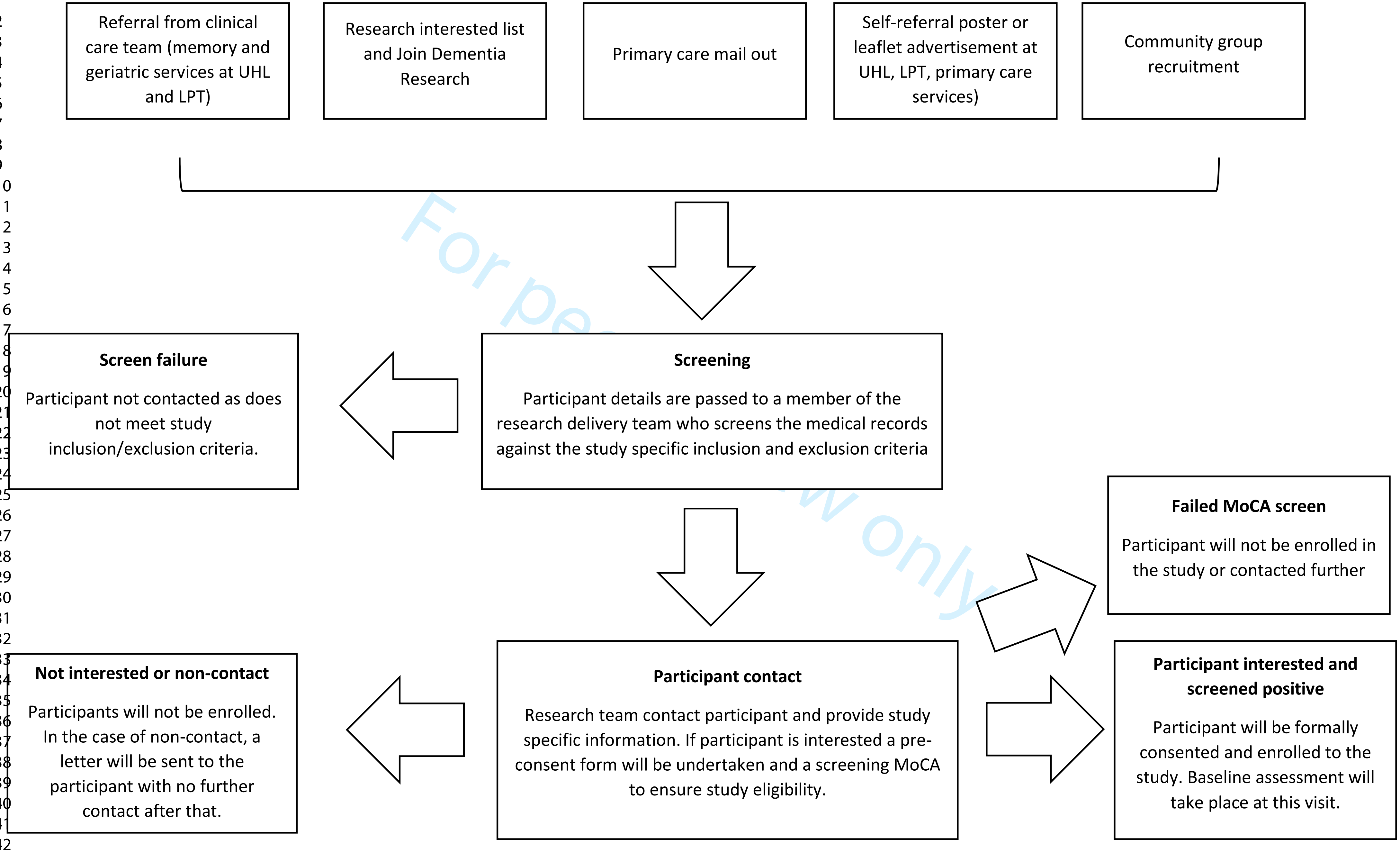
Referral from clinical care team (memory and geriatric services at UHL and LPT)
Research interested list and Join Dementia Research
Primary care mail out
Self-referral poster or leaflet advertisement at UHL, LPT, primary care services)
Community group recruitment
Screen failure
Participant not contacted as does not meet study inclusion/exclusion criteria.
Screening
Participant details are passed to a member of the research delivery team who screens the medical records against the study specific inclusion and exclusion criteria
Failed MoCA screen
Participant will not be enrolled in the study or contacted further
Not interested or non-contact
Participants will not be enrolled. In the case of non-contact, a letter will be sent to the participant with no further contact after that.
Participant contact
Research team contact participant and provide study specific information. If participant is interested a pre-consent form will be undertaken and a screening MoCA to ensure study eligibility.
Participant interested and screened positive
Participant will be formally consented and enrolled to the study. Baseline assessment will take place at this visit.

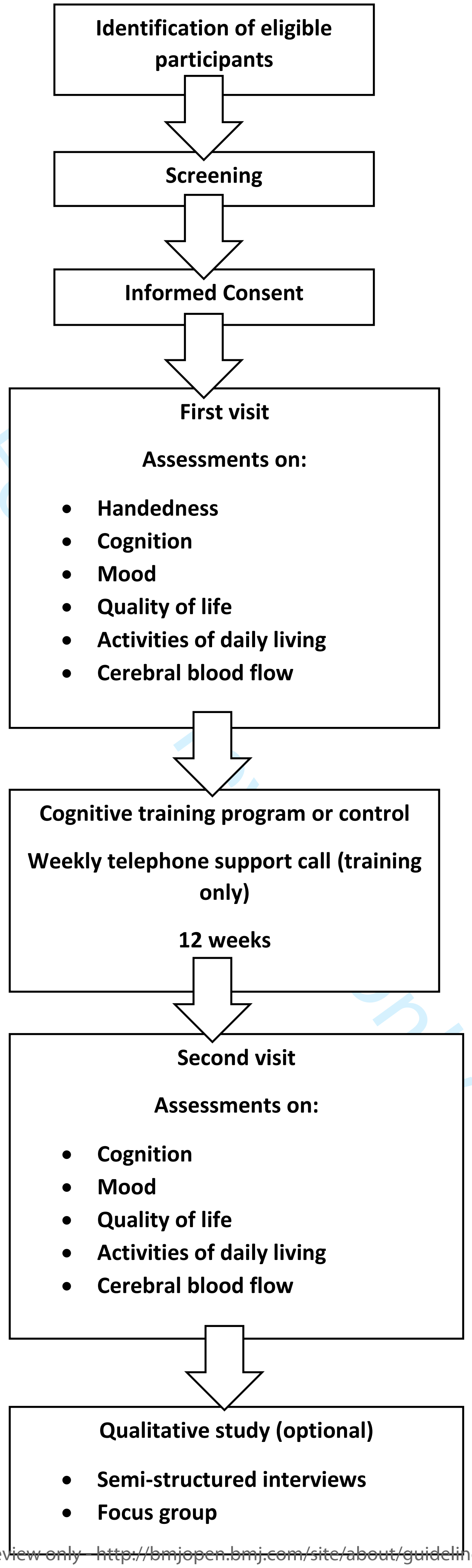

For peer review only - http://bmjopen.bmj.com/site/about/guidelines.xhtml

Supplementary information. Interview and focus group schedule of questions.

Research Questions

1. What are the barriers to brain training in patients with dementia?
2. What are the facilitators (benefits) to brain training in patients with dementia?
3. Could brain training programs be adapted further to support the participation of patients with dementia?
4. Are there any additional benefits to brain training programs not measured by traditional methods as perceived by the patients and their carers?
5. To explore the lived experience of the patient and their carer and the impact brain training has on them and their life

Section 1. Evaluating the Brain Training Program/ Study Design

- How did participants/carers find the program? (opening question)
- What were the positive aspects to completing the program? (RQ2/4)
- What were the negative aspects to completing the program? (RQ1)
- Did any issues or difficulties arise during the program and how did you manage these? (RQ1/2)
- Do you have any suggestions to improve the program for future studies? (RQ 3)

Section 2. Feasibility (RQ3/4)

- Was it difficult to achieve the minimum number of sessions required to complete the program and if yes, why?
- Were the study assessments the right duration?
- Was the frequency of study visits acceptable?
- Would you make any changes to the study visits to make the experience better for people with dementia?

Section 3. Impact on patients and their carers

- How did the program affect your day to day life? (RQ5)
- Did the frequency or duration of the training interfere with your day to day life? (RQ4)
- How do you feel the brain training program affected your mood? (RQ4)
- How do you feel the brain training program affected your memory? (RQ4)
- How do you feel the brain training program affected your ability to carry out day to day activates? (RQ4)

Section 4. The health belief model

1) Risk susceptibility
    a. How likely do you think you are to develop dementia? (healthy volunteers, MCI)
    b. How do you think undertaking a brain training program affects your risk of developing dementia? (HV, MCI)
2) Risk severity

For peer review only

   a. If you were to develop dementia, how serious do you think the consequences would be for you? (HV, MCI)
   b. How severe do you think your memory difficulties are? (AzD, MCI)
   c. How do you feel the brain training program has affected the severity of your symptoms? (AzD, MCI)
3) Benefits to action
   a. What helped you to complete the brain training program? (RQ2)
4) Barriers to action
   a. What stopped you with you completing the brain training program? (RQ1)
5) Self-efficacy
   a. If you experienced difficulties, how did you overcome these to complete the program? (RQ1/2)
6) Cues to action
   a. Did anything support or prompt you to complete the program? (RQ1/2)
   b. Did any thoughts, feelings or symptoms affect you completing the program? (RQ1/2)

For peer review only

Data Security and Storage

Source documents are original documents, data, and records from which participants' CRF data are obtained. These include, but are not limited to, hospital records (from which medical history and previous and concurrent medication may be summarised into the CRF), clinical and office charts, laboratory and pharmacy records, radiographs/scan reports, and correspondence, interview and focus group recordings and transcripts.

CRF entries will be considered source data if the CRF is the site of the original recording (e.g., there is no other written or electronic record of data). In this study the CRF will be used as the source document for; cognition (ACE-III), mood (GDS-15), function/ADLs (Lawton-IADL), quality of life (DEMQOL), handedness (Edinburgh Handedness Inventory), and assessments of neurovascular function (TCD), blood pressure, heart rate and $ETCO_2$.

All documents will be stored safely in confidential conditions. On all study-specific documents, other than the signed consent, the participant will be referred to by the study participant number/code, not by name. All parameters (signals) that are collected during the measurement will be saved using a coded filename. The name and other identifying detail will NOT be included in any study data electronic file. All study data will be entered into Microsoft Excel for Windows. All files will be encrypted and stored on a password secured computer/laptop, which will have restricted access to members who are authorised on the authorisation log. All paper documentation will be stored in a locked filing cabinet, in a locked office, on University of Leicester premises that require identification to enter the building. The participants will be identified by a study specific participant number and/or code in any database. The name and any other identifying detail will NOT be included in any study data electronic file.

For peer review only

Reporting checklist for protocol of a clinical trial.

Based on the SPIRIT guidelines.

Instructions to authors

Complete this checklist by entering the page numbers from your manuscript where readers will find each of the items listed below.

Your article may not currently address all the items on the checklist. Please modify your text to include the missing information. If you are certain that an item does not apply, please write "n/a" and provide a short explanation.

Upload your completed checklist as an extra file when you submit to a journal.

In your methods section, say that you used the SPIRIT reporting guidelines, and cite them as:

Chan A-W, Tetzlaff JM, Altman DG, Laupacis A, Gøtzsche PC, Krleža-Jerić K, Hróbjartsson A, Mann H, Dickersin K, Berlin J, Doré C, Parulekar W, Summerskill W, Groves T, Schulz K, Sox H, Rockhold FW, Rennie D, Moher D. SPIRIT 2013 Statement: Defining standard protocol items for clinical trials. Ann Intern Med. 2013;158(3):200-207

		Reporting Item	Page Number
Title	#1	Descriptive title identifying the study design, population, interventions, and, if applicable, trial acronym	1
Trial registration	#2a	Trial identifier and registry name. If not yet registered, name of intended registry	4,15
Trial registration: data set	#2b	All items from the World Health Organization Trial Registration Data Set	n/a
Protocol version	#3	Date and version identifier	2
Funding	#4	Sources and types of financial, material, and other support	17
Roles and responsibilities: contributorship	#5a	Names, affiliations, and roles of protocol contributors	16
Roles and responsibilities:	#5b	Name and contact information for the trial sponsor	2

For peer review only

sponsor contact information			
Roles and responsibilities: sponsor and funder	#5c	Role of study sponsor and funders, if any, in study design; collection, management, analysis, and interpretation of data; writing of the report; and the decision to submit the report for publication, including whether they will have ultimate authority over any of these activities	17
Roles and responsibilities: committees	#5d	Composition, roles, and responsibilities of the coordinating centre, steering committee, endpoint adjudication committee, data management team, and other individuals or groups overseeing the trial, if applicable (see Item 21a for data monitoring committee)	16
Background and rationale	#6a	Description of research question and justification for undertaking the trial, including summary of relevant studies (published and unpublished) examining benefits and harms for each intervention	5
Background and rationale: choice of comparators	#6b	Explanation for choice of comparators	5,6
Objectives	#7	Specific objectives or hypotheses	7
Trial design	#8	Description of trial design including type of trial (eg, parallel group, crossover, factorial, single group), allocation ratio, and framework (eg, superiority, equivalence, non-inferiority, exploratory)	5
Study setting	#9	Description of study settings (eg, community clinic, academic hospital) and list of countries where data will be collected. Reference to where list of study sites can be obtained	8
Eligibility criteria	#10	Inclusion and exclusion criteria for participants. If applicable, eligibility criteria for study centres and individuals who will perform the interventions (eg, surgeons, psychotherapists)	9
Interventions: description	#11a	Interventions for each group with sufficient detail to allow replication, including how and when they will be administered	10
Interventions: modifications	#11b	Criteria for discontinuing or modifying allocated interventions for a given trial participant (eg, drug dose change in response to harms, participant request, or improving / worsening disease)	12
Interventions: adherance	#11c	Strategies to improve adherence to intervention protocols, and any procedures for monitoring adherence (eg, drug tablet return;	10,11

For peer review only

		laboratory tests)	
Interventions: concomitant care	#11d	Relevant concomitant care and interventions that are permitted or prohibited during the trial	9
Outcomes	#12	Primary, secondary, and other outcomes, including the specific measurement variable (eg, systolic blood pressure), analysis metric (eg, change from baseline, final value, time to event), method of aggregation (eg, median, proportion), and time point for each outcome. Explanation of the clinical relevance of chosen efficacy and harm outcomes is strongly recommended	11,12
Participant timeline	#13	Time schedule of enrolment, interventions (including any run-ins and washouts), assessments, and visits for participants. A schematic diagram is highly recommended (see Figure)	11
Sample size	#14	Estimated number of participants needed to achieve study objectives and how it was determined, including clinical and statistical assumptions supporting any sample size calculations	13,14
Recruitment	#15	Strategies for achieving adequate participant enrolment to reach target sample size	8
Allocation: sequence generation	#16a	Method of generating the allocation sequence (eg, computer-generated random numbers), and list of any factors for stratification. To reduce predictability of a random sequence, details of any planned restriction (eg, blocking) should be provided in a separate document that is unavailable to those who enrol participants or assign interventions	10,11
Allocation concealment mechanism	#16b	Mechanism of implementing the allocation sequence (eg, central telephone; sequentially numbered, opaque, sealed envelopes), describing any steps to conceal the sequence until interventions are assigned	10,11
Allocation: implementation	#16c	Who will generate the allocation sequence, who will enrol participants, and who will assign participants to interventions	10,11
Blinding (masking)	#17a	Who will be blinded after assignment to interventions (eg, trial participants, care providers, outcome assessors, data analysts), and how	11
Blinding (masking): emergency	#17b	If blinded, circumstances under which unblinding is permissible, and procedure for revealing a participant's allocated intervention during	11

For peer review only

unblinding		the trial	
Data collection plan	#18a	Plans for assessment and collection of outcome, baseline, and other trial data, including any related processes to promote data quality (eg, duplicate measurements, training of assessors) and a description of study instruments (eg, questionnaires, laboratory tests) along with their reliability and validity, if known. Reference to where data collection forms can be found, if not in the protocol	11,12
Data collection plan: retention	#18b	Plans to promote participant retention and complete follow-up, including list of any outcome data to be collected for participants who discontinue or deviate from intervention protocols	10,11
Data management	#19	Plans for data entry, coding, security, and storage, including any related processes to promote data quality (eg, double data entry; range checks for data values). Reference to where details of data management procedures can be found, if not in the protocol	12
Statistics: outcomes	#20a	Statistical methods for analysing primary and secondary outcomes. Reference to where other details of the statistical analysis plan can be found, if not in the protocol	13,14
Statistics: additional analyses	#20b	Methods for any additional analyses (eg, subgroup and adjusted analyses)	14
Statistics: analysis population and missing data	#20c	Definition of analysis population relating to protocol non-adherence (eg, as randomised analysis), and any statistical methods to handle missing data (eg, multiple imputation)	13,14
Data monitoring: formal committee	#21a	Composition of data monitoring committee (DMC); summary of its role and reporting structure; statement of whether it is independent from the sponsor and competing interests; and reference to where further details about its charter can be found, if not in the protocol. Alternatively, an explanation of why a DMC is not needed	16
Data monitoring: interim analysis	#21b	Description of any interim analyses and stopping guidelines, including who will have access to these interim results and make the final decision to terminate the trial	16
Harms	#22	Plans for collecting, assessing, reporting, and managing solicited and spontaneously reported adverse events and other unintended effects of trial interventions or trial conduct	16
Auditing	#23	Frequency and procedures for auditing trial conduct, if any, and	n/a

For peer review only

		whether the process will be independent from investigators and the sponsor	
Research ethics approval	#24	Plans for seeking research ethics committee / institutional review board (REC / IRB) approval	16
Protocol amendments	#25	Plans for communicating important protocol modifications (eg, changes to eligibility criteria, outcomes, analyses) to relevant parties (eg, investigators, REC / IRBs, trial participants, trial registries, journals, regulators)	16
Consent or assent	#26a	Who will obtain informed consent or assent from potential trial participants or authorised surrogates, and how (see Item 32)	8
Consent or assent: ancillary studies	#26b	Additional consent provisions for collection and use of participant data and biological specimens in ancillary studies, if applicable	n/a
Confidentiality	#27	How personal information about potential and enrolled participants will be collected, shared, and maintained in order to protect confidentiality before, during, and after the trial	12
Declaration of interests	#28	Financial and other competing interests for principal investigators for the overall trial and each study site	16
Data access	#29	Statement of who will have access to the final trial dataset, and disclosure of contractual agreements that limit such access for investigators	16
Ancillary and post trial care	#30	Provisions, if any, for ancillary and post-trial care, and for compensation to those who suffer harm from trial participation	n/a
Dissemination policy: trial results	#31a	Plans for investigators and sponsor to communicate trial results to participants, healthcare professionals, the public, and other relevant groups (eg, via publication, reporting in results databases, or other data sharing arrangements), including any publication restrictions	15
Dissemination policy: authorship	#31b	Authorship eligibility guidelines and any intended use of professional writers	n/a
Dissemination policy: reproducible research	#31c	Plans, if any, for granting public access to the full protocol, participant-level dataset, and statistical code	n/a
Informed consent materials	#32	Model consent form and other related documentation given to participants and authorised surrogates	n/a

For peer review only

Biological specimens	#33	Plans for collection, laboratory evaluation, and storage of biological specimens for genetic or molecular analysis in the current trial and for future use in ancillary studies, if applicable	n/a

The SPIRIT checklist is distributed under the terms of the Creative Commons Attribution License CC-BY-ND 3.0. This checklist was completed on 08. November 2018 using http://www.goodreports.org/, a tool made by the EQUATOR Network in collaboration with Penelope.ai

For peer review only

The Effects of Brain Training on Brain Blood Flow (The Cognition and Flow Study -CogFlowS): A Protocol for A Feasibility Randomised Controlled Trial of Cognitive Training in Dementia.

Journal:	*BMJ Open*
Manuscript ID	bmjopen-2018-027817.R2
Article Type:	Protocol
Date Submitted by the Author:	11-Apr-2019
Complete List of Authors:	Beishon, Lucy; University of Leicester College of Medicine Biological Sciences and Psychology,<br>Evley, Rachel<br>Panerai, Ronney<br>Subramaniam, Hari<br>Mukaetova-Ladinska, Elizabeta<br>Robinson, Thompson; University of Leicester College of Medicine Biological Sciences and Psychology, Cardiovascular Sciences<br>Haunton, Victoria
**Primary Subject Heading**:	Mental health
Secondary Subject Heading:	Cardiovascular medicine
Keywords:	Neuroimaging, Cerebral haemodynamics, Alzheimer's disease, Mild cognitive impairment, Transcranial Doppler ultrasonography, cognitive impairment

Title

The Effects of Brain Training on Brain Blood Flow (The Cognition and Flow Study -CogFlowS): A Protocol for A Feasibility Randomised Controlled Trial of Cognitive Training in Dementia.

Authors

Lucy Beishon[a], Rachel Evley[b] Ronney B Panerai[a,c], , Hari Subramaniam[d], Elizabeta Mukaetova-Ladinska[d,e], Thompson G Robinson[a,b], Victoria J Haunton[a,b].

Affiliations

[a] University of Leicester, Department of Cardiovascular Sciences, Leicester, UK

[b] Division of Clinical Neuroscience, University of Nottingham, UK

[c] NIHR Leicester Biomedical Research Centre, British Heart Foundation Cardiovascular Research Centre, Glenfield Hospital, Leicester, UK

[d] The Evington Centre, Leicestershire Partnership NHS Trust, Leicester, UK

[e] University of Leicester, Department of Neuroscience, Psychology and Behaviour, Leicester, UK

Corresponding Author

Lucy Beishon

Lb330@le.ac.uk

Room 228, Level 2 Clinical Sciences Building

Leicester Royal Infirmary

Leicester

LE2 7LX

For peer review only

For peer review only - http://bmjopen.bmj.com/site/about/guidelines.xhtml

Tel: 0116 252 3134

Key words

Cognitive impairment

Neuroimaging

Cerebral haemodynamics

Alzheimer's disease

Mild cognitive impairment

Transcranial Doppler ultrasonography

Word count

3679

Protocol date and version number

30/10/2018, version 2

Trial sponsor

Dr Michelle Meussel

Research Governance Office, Leicester General Hospital

Leicester

LE5 4PW

01162584077

uolsponsor@le.ac.uk

For peer review only

Abstract

Introduction

Cognitive training is an emerging non-pharmacological treatment to improve cognitive and physical function in mild cognitive impairment (MCI) and early Alzheimer's disease (AD). Abnormal brain blood flow is a key process in the development of cognitive decline. However, no studies have explored the effects of cognitive training on brain blood flow in dementia. The primary aim of this study is to assess the feasibility for a large scale, randomised controlled trial of cognitive training in healthy older adults (HC), MCI, and early AD.

Methods and analysis

This study will recruit 60 participants, in three sub-groups of twenty (MCI, HC, AD), from primary, secondary, and community services. Participants will be randomised to a twelve week computerised cognitive training programme (five x 30 minute sessions per week), or waiting-list control. Participants will undergo baseline and follow-up assessments of: mood, cognition, quality of life, and activities of daily living. Cerebral blood flow will be measured at rest and during task activation (pre- and post-training) by bilateral transcranial Doppler ultrasonography, alongside heart rate (3-lead ECG), end-tidal CO_2 (capnography), and beat-to-beat blood pressure (Finometer). Participants will be offered to join a focus group or semi-structured interview to explore barriers and facilitators to cognitive training in patients with dementia. Qualitative data will be analysed using framework analysis, and data will be integrated using mixed methods matrices.

Ethics and dissemination

Bradford Leeds Research Ethics committee awarded a favorable opinion (18/YH/0396). Results of the study will be published in peer reviewed journals, and presented at national and international conferences on ageing and dementia.

For peer review only - http://bmjopen.bmj.com/site/about/guidelines.xhtml

Registration

ClinicalTrials.Gov unique identifier: NCT03656107.

Article summary: Strengths and limitations (5 bullet points)

- This study uses a mixed methods approach to investigate the feasibility of cognitive training in healthy older adults, mild cognitive impairment, and Alzheimer's disease
- This study includes an assessment of the effects of cognitive training on neurovascular function, by transcranial Doppler ultrasonography
- This study includes a broad range of outcome measures to take a holistic approach to evaluating cognitive training in dementia
- The primary aim of this study is to identify the feasibility of this protocol in patients with cognitive impairment, and as such is not a fully powered trial
- This study is limited to identifying these outcomes in healthy older adults, mild cognitive impairment, and Alzheimer's disease with mild to moderate deficits

For peer review only

Introduction

The incidence of dementia is rising as the population ages, with 46.8 million people living with dementia worldwide (1, 2). By 2030, this is projected to rise to 75 million (1, 2). However, there are currently few diagnostic or therapeutic strategies available to offer these patients. Dementia is a progressive condition characterised by gradual loss of cognitive and/or non-cognitive higher functions (i.e. language, visuospatial, attention) (3). Mild cognitive impairment (MCI) is characterised by subjective and objective decline in cognitive function, but with preserved functional independence in daily living (4). It has become increasingly recognised that deranged vascular function is an early contributor to the deposition of amyloid plaques and tau tangles in Alzheimer's disease (AD), and that these pathologies exacerbate one another (two-hit hypothesis) (5). In a recent systematic review and meta-analysis, we demonstrated clear abnormalities in vascular function across a number of imaging modalities at the MCI stage (6). Thus, treatments that can improve cerebral perfusion or vascular function could represent an early treatment option for dementia (6). In this study, the target populations are: healthy older adults (as controls), adults with AD, and MCI, to capture the effects of cognitive training on vascular physiology at an early stage in the dementia process.

The Cognition and Flow Study (CogFlowS) is a feasibility, parallel, randomised controlled trial (RCT) to examine the use of a cognitive training programme in adults with MCI, early AD and healthy older adults (HC). In addition to measuring cognitive and functional outcomes, this study will also use transcranial Doppler ultrasonography (TCD) to measure changes in cerebral blood flow (CBF) before and after a 12-week cognitive training program.

Cognitive interventions can be considered as three broad categories: cognitive training, cognitive rehabilitation and cognitive stimulation (7, 8). Cognitive training describes a structured, guided program of standardised tasks designed to provide practice or training within a specified cognitive domain(s), to translate into functional benefits (7, 8). This is distinguished from cognitive rehabilitation, where patient centered approaches and goals are used to improve everyday function,

For peer review only - http://bmjopen.bmj.com/site/about/guidelines.xhtml

rather than improve cognition function (7-9), and cognitive stimulation which aims to generally improve cognition, social function and quality of life (10). Cognitive training is attractive in that it offers a cost effective, non-invasive and acceptable intervention to patients, with no reported adverse effects (11). However, high quality RCT evidence is lacking in studies of cognitive training in dementia (8, 9, 12, 13). Two previous Cochrane reviews (8, 9), highlighted that the evidence was sparse, with many of the studies under-powered, of low-to-moderate quality, and with relatively few for MCI (8, 9). Recent systematic reviews of cognitive training in MCI have demonstrated moderate benefits in verbal learning and memory, global cognition, non-verbal learning, working memory, attention and psychosocial functioning (12, 14). Thus far, studies of cognitive training in dementia have been heterogeneous in study design, participants included, type and definition of training, outcome measures, and duration and intensity of intervention (8, 9, 13, 15, 16). This hampers the adequate meta-analysis of data, and of conclusions that can be drawn from pooled analyses (13, 17). Few studies have included neuroimaging outcomes to explore brain plasticity or neural mechanisms underlying changes in cognition after cognitive training (17). Therefore, the CogFlowS study not only addresses the development of a future high quality RCT of cognitive training in dementia, but also aims to fill the evidence gap on the use of TCD to assess neurovascular plasticity.

TCD is a non-invasive, ultrasound based imaging modality that allows measurements of CBF velocity (CBFv) at rest (18) and following activation by cognitive tasks (19). TCD has a number of advantages over other functional imaging techniques, such as: magnetic resonance imaging (MRI) and positron emission tomography (PET), including lower cost, portability, relative ease of operator training, high temporal resolution and higher acceptability to patients (20, 21). TCD is particularly suited to older people, and those with dementia, as it can be used in patients with pacemakers, metal implants and claustrophobia (20). The use of TCD is a novel method to investigate neurovascular training effects and for markers predictive of which patients may benefit from particular cognitive training programmes. This would allow the development of a personalised approach to developing cognitive training programmes for people with dementia.

For peer review only - http://bmjopen.bmj.com/site/about/guidelines.xhtml

The use of neuroimaging outcomes has also been limited in cognitive training studies (17, 22), but understanding the training effects occurring at the physiological level may provide important mechanistic information to any potential benefits conferred by cognitive training (23-25). Belleville et al demonstrated increased parietal activation in patients with MCI, following a multi-modal cognitive training program designed to improve episodic memory (23). In a near-infrared spectroscopy study by Vermeij et al, patients with MCI had greater training benefits at high load working memory where participants had greater pre-frontal activation (26), whereas baseline global and hippocampal atrophy were predictive of poorer training gains in healthy older adults and those with MCI (24). Therefore, imaging biomarkers can potentially be used to predict and tailor interventions to individual patient's needs (24, 25). Whilst a number of studies have demonstrated positive structural and functional effects of cognitive training (25, 27, 28), they have largely focused on healthy older adults (27), with the use of structural (27) or functional MRI (23, 24) and PET (29, 30); there have been no TCD studies examining changes in neurovascular response to cognitive training in patients with cognitive impairment. In addition to the inclusion of neuroimaging, this study utilises a mixed methods approach. This study includes a qualitative component to explore any benefits, problems or barriers to engaging with the training program, and to identify any benefits not measured by traditional outcomes.

The primary objective for this study is to determine the feasibility for a large scale RCT of cognitive training in HC, AD, and MCI. Secondary objectives seek to identify any clinical benefits of a cognitive training program in terms of: activities of daily living, cognition, mood, and quality of life, and TCD-measured task activation responses in brain blood flow. In addition, the qualitative study seeks to explore barriers and facilitators to the cognitive training programme, how programs can be adapted to support participation for patients living with dementia, and any additional benefits not measured through traditional methods as perceived by patients and their carers. Finally, we plan to explore the lived experience of the patient and their carer, and the impact cognitive training has on them and their life.

For peer review only - http://bmjopen.bmj.com/site/about/guidelines.xhtml

Methods and analysis

This protocol was reported in accordance with the SPIRIT checklist (31), which is included in Supplementary information.

Patient and public involvement

During the design phase of this study, a patient and public involvement (PPI) group was consulted, comprised of research network volunteers from the Alzheimer's society who were either people living with cognitive impairment or their carers. The group reviewed the study design, research questions and outcome measures, and a number of changes were made as a result. These included: providing an option of a focus group or interview to participants, changes to the recruitment procedures, and the design of the cognitive training programme. All study participants will receive a lay summary at the end of the study explaining what the outcomes of the study were. A further PPI meeting was held following successful funding to refine the study documentation and procedures for participants. The trial will be overseen by a trial steering committee (TSC), consisting of patient and public members, and independent academics. The TSC will review the trial conduct on a six monthly basis. As this is a feasibility trial, the burden of intervention is to be assessed in terms of its acceptability to patients and their carers.

Sample selection

HC will be recruited through poster advertisement, friends, family, and carers of enrolled patient participants, Join Dementia Research, and the research interested list at the University of Leicester. Participants with a diagnosis of MCI, or AD, will be recruited through poster advertisement, secondary care services (outpatient clinics within the University Hospitals of Leicester (acute) and the Leicestershire Partnership (mental health) NHS Trusts), research interested lists, Join Dementia Research, and local community groups. In addition, participants will be sought through letter

For peer review only

For peer review only - http://bmjopen.bmj.com/site/about/guidelines.xhtml

invitation from their GP surgery. Participants will be allowed a minimum of 24 hours to decide if they would like to enrol in the study, and to contact the researcher or research delivery team at the Leicestershire Partnership Trust, who will arrange for them to undergo an eligibility assessment, formal consent, and study enrolment. As part of the eligibility assessment participants will undergo a screening cognitive assessment (Montreal Cognitive Assessment – MoCA) to ensure deficits are classified as mild to moderate. Participants will be consented for this, and the MoCA will not form part of the baseline assessments. For participants who lack capacity to consent to the study, a personal consultee declaration will be completed by a friend, relative, or carer, in addition to verbal assent from the participant themselves where possible. Flow charts of the recruitment process and participant procedures can be seen in Figures 1 and 2, respectively.

The planned recruitment target for this study is 60 participants, divided into three sub-groups of twenty (HC, AD, and MCI). As the primary aim of this study is feasibility, a formal sample size calculation has not been performed. Recruiting between 24 and 50 participants for a feasibility study is acceptable and therefore the planned target of 60 participants is sufficiently large enough whilst giving a margin for drop-outs and loss to follow-up (32, 33).

The following inclusion and exclusion criteria were designed to identify a sample of participants that will be able to engage with a cognitive training programme, and to exclude any major medical co-morbidities that can affect CBF:

Inclusion criteria

1) Healthy controls will be free of any medical co-morbidity or medication that could adversely affect cognition. Volunteers with well-controlled co-morbidities (i.e. hypertension, diabetes, will be considered for inclusion)
2) MCI as defined by NIA/AA 2011 (34) and Petersen criteria (4)

For peer review only - http://bmjopen.bmj.com/site/about/guidelines.xhtml

3) AD as defined by the NIA/AA 2011 criteria (34)

And:

4) Deficits will be mild to moderate as defined by Montreal Cognitive Assessment (MoCA) score of >9 for participants with MCI and AD (35-37).

5) Willing to participate

6) Capacity to consent to the study/personal consultee

7) Patients on and off anti-dementia medications will be included (acetylcholinesterase inhibitors, glutamate receptor antagonists)

8) Good understanding of written and spoken English

9) Age >50 years

10) Access to the internet and a computer/laptop device.

Exclusion criteria

1) Healthy controls with any medical co-morbidity or medication that could adversely affect cognition, or poorly controlled medical co-morbidities (i.e. hypertension, diabetes)

2) Unwilling to take part

3) Unable to consent/no personal consultee

4) Major medical co-morbidity: severe heart failure (ejection fraction <20%), carotid artery stenosis, severe respiratory disease, major stroke

5) Pregnancy, planning pregnancy, or lactating

6) Inadequate bilateral TCD windows

7) Participants already enrolled into other interventional studies that would confound study results

8) Insufficient understanding of written and spoken English

For peer review only - http://bmjopen.bmj.com/site/about/guidelines.xhtml

9) Age <50 years

10) No access to the internet and a computer/laptop device

Intervention

Participants randomised to the intervention will complete a twelve week cognitive training programme. Participants will be required to complete five, 30 minute sessions per week for twelve weeks. The cognitive training programme will be provided by Lumosity© as part of a collaboration through the Human Cognition Project. Lumosity© is commercially available software, developed by a group of neuropsychologists, which has been used across several studies of brain training and disciplines (38-40). These studies have been included in a number of systematic reviews and meta-analysis (12, 41). It is a multi-domain, online-based cognitive training tool, which is relatively easy to use and administer. It has been designed to adapt to the individual's cognitive function in order to administer the training program to their individual level of cognitive function. Exercises will be selected from Lumosity's© commercially available online software which have been pre-classified to target the following cognitive domains: attention, memory, visuospatial, verbal fluency, and language. These five cognitive domains will be evaluated through formal cognitive testing, and through the neurovascular coupling assessment. Compliance will be monitored through Lumosity© online software which will log and track the number of minutes and times per week a participant has spent training, in order to calculate the dose of training to which each participant has been exposed.

Randomisation

Randomisation will be performed using Sealed Envelope©, by the researcher. This is an online-based randomisation tool which uses random permuted blocks to allocate participants to waiting list control or intervention. Participants will be enrolled and assigned a PIN consecutively, and randomised to a treatment arm corresponding to the PIN. Given the nature of the trial, it is not possible to blind participants to the intervention. The Investigator will be providing weekly telephone support for the intervention group, in addition to undertaking baseline and follow-up measurements, and therefore

For peer review only - http://bmjopen.bmj.com/site/about/guidelines.xhtml

blinding of the investigator is also not possible. However, data analysis will be blinded by generating a batch-anonymised data set. Randomisation will be undertaken at the initial visit. Once participants have been randomised to the intervention arm, they will be provided with information on how to access and use the cognitive training programme at home.

Data collection and integration

All participants (intervention and control groups) will be required to attend two assessments: baseline, and follow-up at twelve weeks post-training. Assessments will be carried out at the participant's home, or designated research space at the Leicestershire Partnership Trust, or Cerebral Haemodynamics in Ageing and Stroke Medicine (CHiASM) research space. Participants will been given the option to divide the assessments across two visits if necessary. This is to reduce any burden of participation for people living with dementia, who may fatigue more quickly with the baseline and follow-up assessments. It is anticipated that the majority of participants will be able to complete all assessments in one visit, and this will be included within the feasibility assessment for the study.

Data will be collected on demographics, medical history, and medication use. Assessments of: cognition [Addenbrooke's cognitive examination III (ACE-III)], mood (Geriatric Depression Scale 15), activities of daily living (Lawton instrumental activities of daily living), and quality of life (Dementia Rated Quality of Life), will be carried out at both visits. An assessment of neurovascular function will be carried out at the CHiASM research space, a temperature controlled room, free of noise and distraction. Participants will be seated throughout the protocol. All measurements will be made at rest (five minute baseline recording), and during cerebral activation (selected cognitive tasks). CBFv will be measured continuously by insonation of the bilateral middle cerebral arteries (MCA) (DWL Doppler Box), using 2 MHz probes, fixed in place with a headframe. Beat-to-beat blood pressure will be measured continuously using a Finometer cuff on the non-dominant middle finger (Finapres Medical Systems, Amsterdam), and calibrated using a brachial blood pressure recording (UA767 BP monitor). End-tidal CO_2 will be measured by capnography using nasal cannulae (Capnochek Plus), and

For peer review only - http://bmjopen.bmj.com/site/about/guidelines.xhtml

the R-R interval will be recorded using a 3-lead ECG. The task activation protocol has been published previously (42, 43). In brief, participants will be presented with five tasks selected from the ACE-III, and percentage increase in CBFv from resting baseline will be calculated at task initiation. The tasks will be selected for five cognitive domains: attention, memory, visuospatial, language, and verbal fluency, based on recent studies conducted by this group (43, 44). Data will be collected on the following parameters: average CBFv, $ETCO_2$, HR, and BP at rest over five minutes, and peak percentage change from baseline in all parameters at 5-10 seconds, and 10-20 seconds after task activation for each of the five cognitive tasks.

At least one week after completion of the follow-up assessment, participants with a diagnosis of MCI, or AD, will be invited to attend a semi-structured interview or focus group. The schedule of questions can be seen in supplementary information. The schedule of questions has been framed around the six constructs of the health belief model (risk susceptibility, risk severity, benefits to action, barriers to action, self-efficacy, and cues to action) (45) to evaluate the cognitive training programme, and the experience of the participant and their carer. Interviews and focus groups will be recorded continuously with a digital audio recorder, and notes of non-verbal and paralinguistic clues will be made. All digital recordings will be transcribed within five to seven days of the interview or focus group. Semi-structured interviews are an iterative process, following the first couple of interviews the transcripts will be analysed and the themes and concepts emerging from these initial data will be further explored at the following interviews.

Data security and storage will be conducted in line with general data protection regulations (https://eugdpr.org/), and details regarding data security and storage can be seen in Supplementary information.

Trial discontinuation

For peer review only - http://bmjopen.bmj.com/site/about/guidelines.xhtml

This is not a drug intervention trial, and therefore interim analysis will not be required. Criteria for trial discontinuation are as follows: ineligibility, significant protocol deviation, significant non-adherence to the programme, an adverse event or disease progression resulting in inability to comply with study procedures, consent withdrawn, or lost to follow-up.

Data analysis and interpretation

The difference for each participant between baseline and follow-up assessment for each of the continuous outcome measures described above will be reported along with the overall mean difference.

CBFv data will be recorded and stored in the PHYSIDAS system, and analysed offline. Data will be visually inspected for quality, and files rejected where quality is deemed to be poor, with reasons. Peak percentage change in CBFv will be calculated relative to a twenty second baseline, prior to task initiation.

The findings from the semi-structured interviews and focus groups will be evaluated using framework analysis (41). The digital recordings of the interviews and focus groups will be transcribed verbatim, the transcripts will be read in detail, i.e. line-by-line, and open codes will be formed categorising and conceptualising the responses and identifying the major themes. Two researchers LB & RE will independently code the first few transcripts to ensure consistency in coding.

Following this initial coding the analytical framework will be developed, this is an iterative process and will develop through coding of additional transcripts. Once the final transcript has been coded the analytic framework will be used to generate the framework matrix. The framework matrix will be developed in NVivo 11 (QSR International), and allow for the recognition of patterns and outliers within the data. Respondent validation will be used to establish a degree of correspondence between the researcher's views and those of the research participant (46).

For peer review only - http://bmjopen.bmj.com/site/about/guidelines.xhtml

Data from the quantitative and qualitative arms of the study will be integrated using mixed methods approaches of triangulation and mixed methods matrices (47).

Statistical analysis and sample size calculation

Data will be checked for normality, and non-normally distributed data will be appropriately transformed, and parametric tests applied. Data will be reported as mean (standard deviation) for continuous variables, and number (percentage) for nominal data. Differences in baseline demographics between the intervention and control groups will be analysed within population group (HC/AD/MCI) using Chi-Square for nominal data, and independent t-testing for continuous data. Any baseline differences in demographics between population groups will also be reported. To assess the impact of the intervention and population group on each of these differences, a two-way ANOVA will be carried out. The interaction between population group and treatment group will be analysed to assess if the difference between the treatment groups is different between the population groups. Post-hoc testing will be conducted by Tukey to analyse for main effects. Data will be recorded in Microsoft Excel. Statistical analyses will be performed using the latest version of SPSS for Windows (SPSS v.24), and graphs will be produced using the latest version of GraphPad Prism for Windows.

Study limitations

This is a feasibility study, and thus will not be powered adequately to detect changes in the secondary end points outlined above. However, given the novel aspects of this study protocol, such as the inclusion of a TCD-measured changes in CBF responses, and the mixed methods design, this requires evaluation of the feasibility before moving to a larger, fully powered trial of cognitive training in dementia. Furthermore, this study will include assessments of patient experience and tolerability to facilitate this. Although TCD affords excellent temporal resolution in the measurement of CBFv, the MCA supplies blood to approximately 80% of the cerebral cortex, and therefore reflects a global measure of perfusion, and cannot discriminate where changes are occurring, due to poor spatial resolution. Techniques such as MRI, and PET scans afford better spatial resolution, but have their own

For peer review only - http://bmjopen.bmj.com/site/about/guidelines.xhtml

For peer review only

limitations, particularly in older patients with cognitive deficits. TCD-measured CBFv relies on the assumption that the vessel diameter remains constant, despite fluctuations in CO_2 and blood pressure (18). This study elected to focus on healthy older adults, MCI and AD in the first instance. Deficits in CBFv have been demonstrated in both MCI and AD, but inclusion of participants with vascular cognitive impairment would provide further information on the capacity for plasticity to neurovascular physiology amongst dementia sub-types. In addition, only participants with mild to moderate deficits will be included, given that data thus far do not support the use of cognitive training in more advanced dementia (9, 12). Only participants who are undertaking the training programme will be offered telephone support, as it is primarily to support technical issues with delivery of the programme. However, this could introduce a placebo effect, and future studies would ideally correct for this by providing support to both groups. Finally, one investigator is undertaking both baseline and follow-up assessments, and providing telephone support, which carries a risk of introducing researcher-bias into the study. As this is primarily a feasibility study, this is of lesser importance here, but a future trial should ideally have separate team members conducting these different roles to minimise the risk of bias.

Ethics and dissemination

Ethics and safety considerations

This study has been reviewed by the Bradford Leeds Research Ethics Committee (REC), and was granted a favorable opinion on the 7/11/18 (18/YH/0396). All participants with capacity in this study will provide informed, written consent, and the study will be conducted in accordance with the Declaration of Helsinki and Good Medical Practice guidelines (48). Given the nature of dementia, not all participants will have capacity to consent to the study. In this instance, a personal consultee (friend, relative, or carer) will be consulted for their opinion on whether the participant is likely to have wanted to participate in this study, and they will be asked to sign a consultee declaration form. It was not

For peer review only - http://bmjopen.bmj.com/site/about/guidelines.xhtml

considered ethical to exclude participants on the grounds of capacity, given the nature of the disease studied.

Dissemination

The results from this study will be submitted to peer-reviewed journals in ageing and dementia research, and presented at national and international conferences on these themes. We anticipate the results will be presented and published in late 2020, early 2021. In addition, the results will be disseminated to members of the community through the Alzheimer's Society, community groups, podcasts, and social media.

Trial registration

This study has been registered on ClinicalTrials.gov (ref: NCT03656107). The trial was registered prior to initiation, and will be completed within six months of recruitment of the first study participant.

Ethics approval

Ethical approval was obtained from the Bradford Leeds Research Ethics Committee (18/YH/0396)

Competing interests

None.

Author Contributions

The quantitative arm of the study was conceived and designed by LB, VJH, RBP, and TGR. LB and RE designed the qualitative arm of the study. HS and EML reviewed and edited the protocol. All authors assisted in drafting this manuscript.

Trial committees and monitoring

The study will be monitored at six-monthly intervals throughout its duration by a trial steering committee, independent of the funder, consisting of lay and independent academic members. Each site will have a lead investigator who is responsible for the identification and recruitment of

For peer review only - http://bmjopen.bmj.com/site/about/guidelines.xhtml

For peer review only

participants, data collection and completion for CRFs, follow-up measurements, and adherence to study protocols. Serious adverse events (SAEs) will be reported to the sponsor within one working day of identification, and the Sponsor will report all serious unexpected or adverse reactions to the ethics committee concerned. The chief investigator will submit an annual report to the ethics committee which lists all SAEs. Where necessary, protocol amendments will be submitted to the study sponsor or ethics committee for approval.

Provenance and peer review

Not commissioned.

Data sharing agreement

The data will be stored in the Leicester Research Archive after study completion, and data will be made available at request.

Funding

This study was reviewed and funded by the Dunhill Medical Trust, and LB is a Dunhill research training fellow (RTF1806\27). TGR is an NIHR Senior Investigator. This funding source had and will not have a role in the design, conduct, analyses, interpretation of the data, or decision to submit results. The cognitive training programme is being provided by Lumosity© free of charge, as part of an industry-institution collaboration through the Human Cognition Project. Lumosity© is providing advice and support on the composition and nature of the programme. However, this study is financially independent of Lumosity©, and Lumosity© cannot influence or change the results or outcomes of this study.

Acknowledgments

We would like to thank the members of the patient and public involvement group from the Alzheimer's Society for their contribution to the development of this protocol.

For peer review only - http://bmjopen.bmj.com/site/about/guidelines.xhtml

For peer review only

For peer review only - http://bmjopen.bmj.com/site/about/guidelines.xhtml

Figure Legends

Figure 1. Flow chart of study recruitment procedures.

Figure 2. Flow chart of participant procedures during the study.

For peer review only

References

1. Alzheimer's-society. Demography: Alzheimer's Society; 2016 [updated 2016-11-28. Available from: https://www.alzheimers.org.uk/info/20091/position_statements/93/demography.
2. Alzheimer's-Association. 2014 Alzheimer's disease facts and figures. Alzheimer's & dementia : the journal of the Alzheimer's Association. 2014;10(2):e47-92.
3. Robinson L, Tang E, Taylor JP. Dementia: timely diagnosis and early intervention. BMJ. 2015;350:h3029.
4. Petersen RC. Mild cognitive impairment as a diagnostic entity. J Intern Med. 2004;256(3):183-94.
5. Zlokovic BV. Neurovascular pathways to neurodegeneration in Alzheimer's disease and other disorders. Nat Rev Neurosci. 2011;12(12):723-38.
6. Beishon L, Haunton VJ, Panerai RB, Robinson TG. Cerebral Hemodynamics in Mild Cognitive Impairment: A Systematic Review. J Alzheimers Dis. 2017;59(1):369-85.
7. Belleville S. Cognitive training for persons with mild cognitive impairment. Int Psychogeriatr. 2008;20(1):57-66.
8. Martin M, Clare L, Altgassen AM, Cameron MH, Zehnder F. Cognition-based interventions for healthy older people and people with mild cognitive impairment. The Cochrane database of systematic reviews. 2011(1):Cd006220.
9. Bahar-Fuchs A, Clare L, Woods B. Cognitive training and cognitive rehabilitation for persons with mild to moderate dementia of the Alzheimer's or vascular type: a review. Alzheimers Res Ther. 2013;5(4):35.
10. Woods B, Aguirre E, Spector AE, Orrell M. Cognitive stimulation to improve cognitive functioning in people with dementia. The Cochrane database of systematic reviews. 2012(2):Cd005562.
11. Gates NJ, Sachdev P. Is cognitive training an effective treatment for preclinical and early Alzheimer's disease? J Alzheimers Dis. 2014;42 Suppl 4:S551-9.
12. Hill NT, Mowszowski L, Naismith SL, Chadwick VL, Valenzuela M, Lampit A. Computerized Cognitive Training in Older Adults With Mild Cognitive Impairment or Dementia: A Systematic Review and Meta-Analysis. Am J Psychiatry. 2017;174(4):329-40.
13. Gates NJ, Sachdev PS, Fiatarone Singh MA, Valenzuela M. Cognitive and memory training in adults at risk of dementia: a systematic review. BMC Geriatr. 2011;11:55.
14. Li H, Li J, Li N, Li B, Wang P, Zhou T. Cognitive intervention for persons with mild cognitive impairment: A meta-analysis. Ageing Res Rev. 2011;10(2):285-96.
15. Huntley JD, Gould RL, Liu K, Smith M, Howard RJ. Do cognitive interventions improve general cognition in dementia? A meta-analysis and meta-regression. BMJ Open. 2015;5(4).
16. Reijnders J, van Heugten C, van Boxtel M. Cognitive interventions in healthy older adults and people with mild cognitive impairment: a systematic review. Ageing Res Rev. 2013;12(1):263-75.
17. Coyle H, Traynor V, Solowij N. Computerized and virtual reality cognitive training for individuals at high risk of cognitive decline: systematic review of the literature. Am J Geriatr Psychiatry. 2015;23(4):335-59.
18. Panerai RB. Transcranial Doppler for evaluation of cerebral autoregulation. Clin Auton Res. 2009;19(4):197-211.
19. Stroobant N, Vingerhoets G. Transcranial Doppler ultrasonography monitoring of cerebral hemodynamics during performance of cognitive tasks: a review. Neuropsychol Rev. 2000;10(4):213-31.
20. van Beek AH, Claassen JA, Rikkert MG, Jansen RW. Cerebral autoregulation: an overview of current concepts and methodology with special focus on the elderly. J Cereb Blood Flow Metab. 2008;28(6):1071-85.
21. Tomek A, Urbanova B, Hort J. Utility of transcranial ultrasound in predicting Alzheimer's disease risk. J Alzheimers Dis. 2014;42:S365-S74.

For peer review only - http://bmjopen.bmj.com/site/about/guidelines.xhtml

22. Vemuri P, Fields J, Peter J, Klöppel S. Cognitive interventions in Alzheimer's and Parkinson's diseases: emerging mechanisms and role of imaging. Curr Opin Neurol. 2016;29(4):405-11.
23. Belleville S, Clement F, Mellah S, Gilbert B, Fontaine F, Gauthier S. Training-related brain plasticity in subjects at risk of developing Alzheimer's disease. Brain. 2011;134(Pt 6):1623-34.
24. Vermeij A, Claassen JA, Dautzenberg PL, Kessels RP. Transfer and maintenance effects of online working-memory training in normal ageing and mild cognitive impairment. Neuropsychol Rehabil. 2016;26(5-6):783-809.
25. Belleville S, Bherer L. Biomarkers of Cognitive Training Effects in Aging. Curr Transl Geriatr Exp Gerontol Rep. 2012;1(2):104-10.
26. Vermeij A, Kessels RPC, Heskamp L, Simons EMF, Dautzenberg PLJ, Claassen JAHR. Prefrontal activation may predict working-memory training gain in normal aging and mild cognitive impairment. Brain Imaging and Behavior. 2016:1-14.
27. Engvig A, Fjell AM, Westlye LT, Moberget T, Sundseth O, Larsen VA, et al. Effects of memory training on cortical thickness in the elderly. Neuroimage. 2010;52(4):1667-76.
28. Yang H, Leaver AM, Siddarth P, Paholpak P, Ercoli L, St. Cyr NM, et al. Neurochemical and Neuroanatomical Plasticity Following Memory Training and Yoga Interventions in Older Adults with Mild Cognitive Impairment. Front Aging Neurosci. 2016;8.
29. Ciarmiello A, Gaeta MC, Benso F, Del Sette M. FDG-PET in the Evaluation of Brain Metabolic Changes Induced by Cognitive Stimulation in aMCI Subjects. Curr Radiopharm. 2015;8(1):69-75.
30. Forster S, Buschert VC, Teipel SJ, Friese U, Buchholz HG, Drzezga A, et al. Effects of a 6-month cognitive intervention on brain metabolism in patients with amnestic MCI and mild Alzheimer's disease. J Alzheimers Dis. 2011;26 Suppl 3:337-48.
31. Chan AW, Tetzlaff JM, Altman DG, Laupacis A, Gøtzsche PC, Krleža-Jerić K, et al. SPIRIT 2013 Statement: Defining Standard Protocol Items for Clinical Trials. Ann Intern Med. 2013;158(3):200-7.
32. Sim J, Lewis M. The size of a pilot study for a clinical trial should be calculated in relation to considerations of precision and efficiency. J Clin Epidemiol. 2012;65(3):301-8.
33. Julious SA. Sample sizes for clinical trials with normal data. Stat Med. 2004;23(12):1921-86.
34. Albert MS, DeKosky ST, Dickson D, Dubois B, Feldman HH, Fox NC, et al. The diagnosis of mild cognitive impairment due to Alzheimer's disease: recommendations from the National Institute on Aging-Alzheimer's Association workgroups on diagnostic guidelines for Alzheimer's disease. Alzheimer's & dementia : the journal of the Alzheimer's Association. 2011;7(3):270-9.
35. Saczynski JS, Inouye SK, Guess J, Jones RN, Fong TG, Nemeth E, et al. The Montreal Cognitive Assessment (MoCA): Creating a Crosswalk with the Mini-Mental State Examination. J Am Geriatr Soc. 2015;63(11):2370-4.
36. Borland E, Nagga K, Nilsson PM, Minthon L, Nilsson ED, Palmqvist S. The Montreal Cognitive Assessment: Normative Data from a Large Swedish Population-Based Cohort. J Alzheimers Dis. 2017;59(3):893-901.
37. Tan JP, Li N, Gao J, Wang LN, Zhao YM, Yu BC, et al. Optimal cutoff scores for dementia and mild cognitive impairment of the Montreal Cognitive Assessment among elderly and oldest-old Chinese population. J Alzheimers Dis. 2015;43(4):1403-12.
38. Kesler S, Hadi Hosseini SM, Heckler C, Janelsins M, Palesh O, Mustian K, et al. Cognitive training for improving executive function in chemotherapy-treated breast cancer survivors. Clin Breast Cancer. 2013;13(4):299-306.
39. Finn M, McDonald S. A single case study of computerised cognitive training for older persons with mild cognitive impairment. NeuroRehabilitation. 2014;35(2):261-70.
40. Ballesteros S, Prieto A, Mayas J, Toril P, Pita C, Ponce de León L, et al. Brain training with non-action video games enhances aspects of cognition in older adults: a randomized controlled trial. Front Aging Neurosci. 2014;6.
41. Lampit A, Hallock H, Valenzuela M. Computerized cognitive training in cognitively healthy older adults: a systematic review and meta-analysis of effect modifiers. PLoS Med. 2014;11(11):e1001756.

For peer review only - http://bmjopen.bmj.com/site/about/guidelines.xhtml

42. Beishon L, Williams CAL, Panerai RB, Robinson TG, Haunton VJ. Reproducibility of task activation using the Addenbrooke's cognitive examination in healthy controls: A functional Transcranial Doppler ultrasonography study. J Neurosci Methods. 2017.
43. Beishon LC, Williams CAL, Panerai RB, Robinson TG, Haunton VJ. The assessment of neurovascular coupling with the Addenbrooke's Cognitive Examination: a functional Transcranial Doppler Ultrasonographic Study. J Neurophysiol. 2017:jn.00698.2017.
44. Beishon L, Panerai RB, Robinson TG, Subramaniam H, Haunton VJ. The assessment of cerebrovascular response to a language task from the Addenbrooke's cognitive examination in cognitive impairment: A feasibility functional transcranial Doppler ultrasonography study. Journal of Alzheimer's Disease Reports. 2018;2:153-64.
45. Jones CL, Jensen JD, Scherr CL, Brown NR, Christy K, Weaver J. The Health Belief Model as an Explanatory Framework in Communication Research: Exploring Parallel, Serial, and Moderated Mediation. Health Commun. 2015;30(6):566-76.
46. Hansen EC. Successful Qualitative Health Research: A Practical Introduction: Maidenhead: Open University Press; 2006.
47. O'Cathain A, Murphy E, Nicholl J. Three techniques for integrating data in mixed methods studies. BMJ. 2010;341:c4587.
48. Vijayananthan A, Nawawi O. The importance of Good Clinical Practice guidelines and its role in clinical trials. Biomedical Imaging and Intervention Journal. 2008;4(1).

For peer review only

For peer review only - http://bmjopen.bmj.com/site/about/guidelines.xhtml

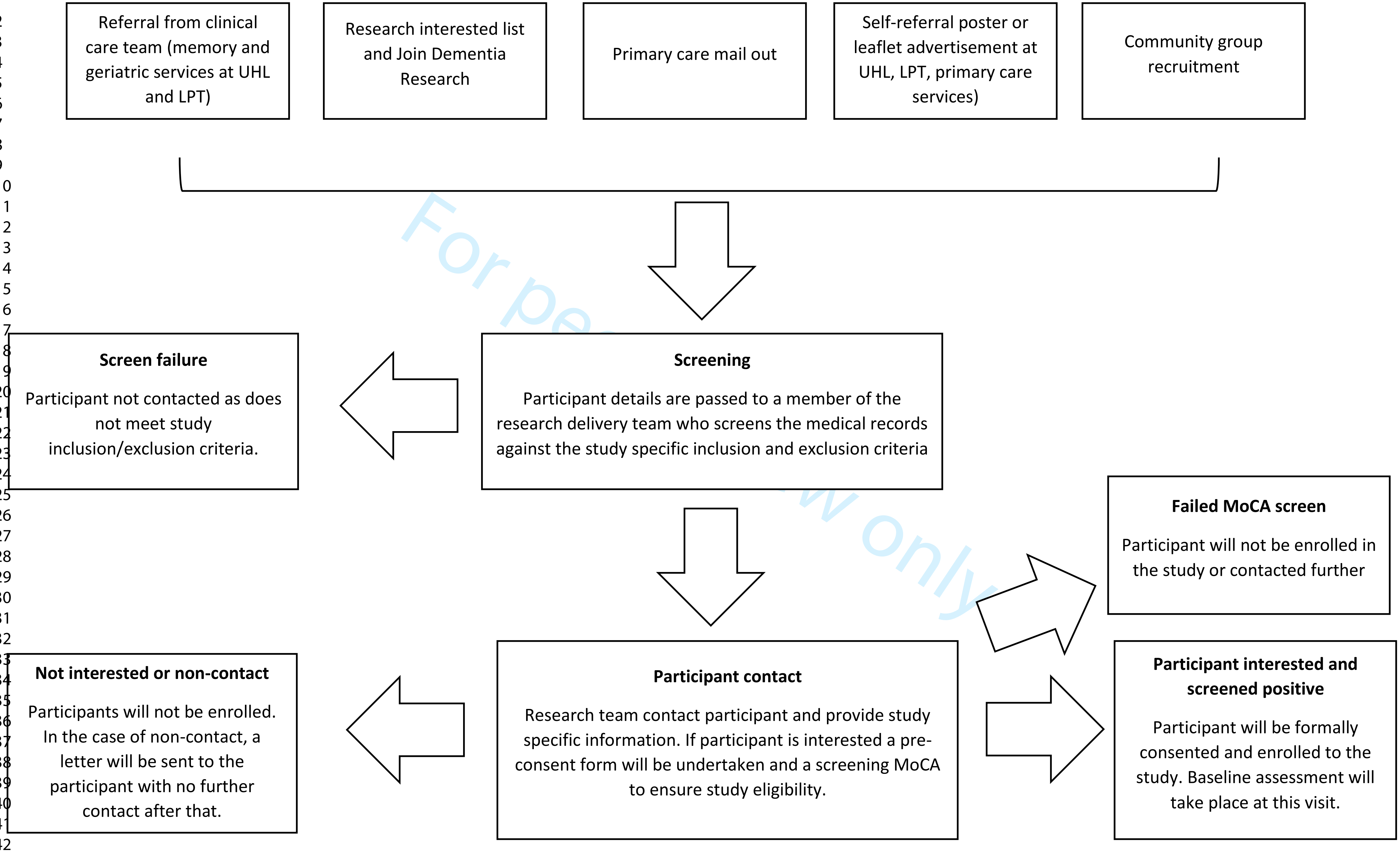
Referral from clinical care team (memory and geriatric services at UHL and LPT)
Research interested list and Join Dementia Research
Primary care mail out
Self-referral poster or leaflet advertisement at UHL, LPT, primary care services)
Community group recruitment
Screen failure
Participant not contacted as does not meet study inclusion/exclusion criteria.
Screening
Participant details are passed to a member of the research delivery team who screens the medical records against the study specific inclusion and exclusion criteria
Failed MoCA screen
Participant will not be enrolled in the study or contacted further
Not interested or non-contact
Participants will not be enrolled. In the case of non-contact, a letter will be sent to the participant with no further contact after that.
Participant contact
Research team contact participant and provide study specific information. If participant is interested a pre-consent form will be undertaken and a screening MoCA to ensure study eligibility.
Participant interested and screened positive
Participant will be formally consented and enrolled to the study. Baseline assessment will take place at this visit.

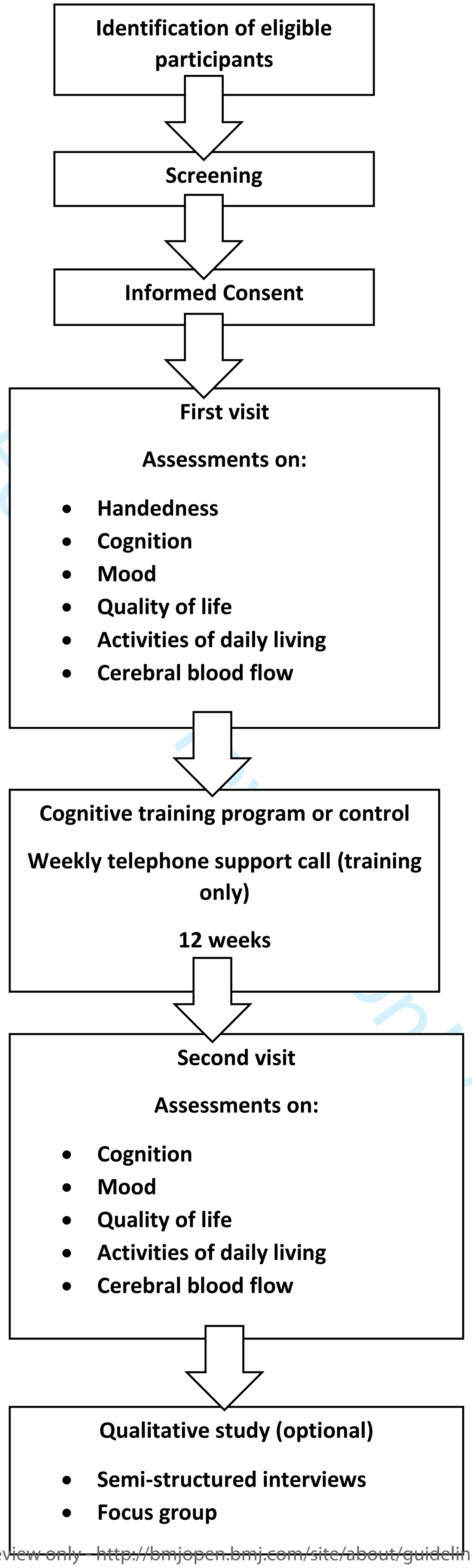
Identification of eligible participants
Screening
Informed Consent
First visit
Assessments on:
Handedness
Cognition
Mood
Quality of life
Activities of daily living
Cerebral blood flow
Cognitive training program or control
Weekly telephone support call (training only)
12 weeks
Second visit
Assessments on:
Cognition
Mood
Quality of life
Activities of daily living
Cerebral blood flow
Qualitative study (optional)
Semi-structured interviews
Focus group

Supplementary information. Interview and focus group schedule of questions.

Research Questions

1. What are the barriers to brain training in patients with dementia?
2. What are the facilitators (benefits) to brain training in patients with dementia?
3. Could brain training programs be adapted further to support the participation of patients with dementia?
4. Are there any additional benefits to brain training programs not measured by traditional methods as perceived by the patients and their carers?
5. To explore the lived experience of the patient and their carer and the impact brain training has on them and their life

Section 1. Evaluating the Brain Training Program/ Study Design

- How did participants/carers find the program? (opening question)
- What were the positive aspects to completing the program? (RQ2/4)
- What were the negative aspects to completing the program? (RQ1)
- Did any issues or difficulties arise during the program and how did you manage these? (RQ1/2)
- Do you have any suggestions to improve the program for future studies? (RQ 3)

Section 2. Feasibility (RQ3/4)

- Was it difficult to achieve the minimum number of sessions required to complete the program and if yes, why?
- Were the study assessments the right duration?
- Was the frequency of study visits acceptable?
- Would you make any changes to the study visits to make the experience better for people with dementia?

Section 3. Impact on patients and their carers

- How did the program affect your day to day life? (RQ5)
- Did the frequency or duration of the training interfere with your day to day life? (RQ4)
- How do you feel the brain training program affected your mood? (RQ4)
- How do you feel the brain training program affected your memory? (RQ4)
- How do you feel the brain training program affected your ability to carry out day to day activates? (RQ4)

Section 4. The health belief model

1) Risk susceptibility
    a. How likely do you think you are to develop dementia? (healthy volunteers, MCI)
    b. How do you think undertaking a brain training program affects your risk of developing dementia? (HV, MCI)
2) Risk severity

For peer review only

a. If you were to develop dementia, how serious do you think the consequences would be for you? (HV, MCI)
b. How severe do you think your memory difficulties are? (AzD, MCI)
c. How do you feel the brain training program has affected the severity of your symptoms? (AzD, MCI)

3) Benefits to action
    a. What helped you to complete the brain training program? (RQ2)
4) Barriers to action
    a. What stopped you with you completing the brain training program? (RQ1)
5) Self-efficacy
    a. If you experienced difficulties, how did you overcome these to complete the program? (RQ1/2)
6) Cues to action
    a. Did anything support or prompt you to complete the program? (RQ1/2)
    b. Did any thoughts, feelings or symptoms affect you completing the program? (RQ1/2)

For peer review only

Data Security and Storage

Source documents are original documents, data, and records from which participants' CRF data are obtained. These include, but are not limited to, hospital records (from which medical history and previous and concurrent medication may be summarised into the CRF), clinical and office charts, laboratory and pharmacy records, radiographs/scan reports, and correspondence, interview and focus group recordings and transcripts.

CRF entries will be considered source data if the CRF is the site of the original recording (e.g., there is no other written or electronic record of data). In this study the CRF will be used as the source document for; cognition (ACE-III), mood (GDS-15), function/ADLs (Lawton-IADL), quality of life (DEMQOL), handedness (Edinburgh Handedness Inventory), and assessments of neurovascular function (TCD), blood pressure, heart rate and $ETCO_2$.

All documents will be stored safely in confidential conditions. On all study-specific documents, other than the signed consent, the participant will be referred to by the study participant number/code, not by name. All parameters (signals) that are collected during the measurement will be saved using a coded filename. The name and other identifying detail will NOT be included in any study data electronic file. All study data will be entered into Microsoft Excel for Windows. All files will be encrypted and stored on a password secured computer/laptop, which will have restricted access to members who are authorised on the authorisation log. All paper documentation will be stored in a locked filing cabinet, in a locked office, on University of Leicester premises that require identification to enter the building. The participants will be identified by a study specific participant number and/or code in any database. The name and any other identifying detail will NOT be included in any study data electronic file.

For peer review only

Reporting checklist for protocol of a clinical trial.

Based on the SPIRIT guidelines.

Instructions to authors

Complete this checklist by entering the page numbers from your manuscript where readers will find each of the items listed below.

Your article may not currently address all the items on the checklist. Please modify your text to include the missing information. If you are certain that an item does not apply, please write "n/a" and provide a short explanation.

Upload your completed checklist as an extra file when you submit to a journal.

In your methods section, say that you used the SPIRIT reporting guidelines, and cite them as:

Chan A-W, Tetzlaff JM, Altman DG, Laupacis A, Gøtzsche PC, Krleža-Jerić K, Hróbjartsson A, Mann H, Dickersin K, Berlin J, Doré C, Parulekar W, Summerskill W, Groves T, Schulz K, Sox H, Rockhold FW, Rennie D, Moher D. SPIRIT 2013 Statement: Defining standard protocol items for clinical trials. Ann Intern Med. 2013;158(3):200-207

		Reporting Item	Page Number
Title	#1	Descriptive title identifying the study design, population, interventions, and, if applicable, trial acronym	1
Trial registration	#2a	Trial identifier and registry name. If not yet registered, name of intended registry	4,15
Trial registration: data set	#2b	All items from the World Health Organization Trial Registration Data Set	n/a
Protocol version	#3	Date and version identifier	2
Funding	#4	Sources and types of financial, material, and other support	17
Roles and responsibilities: contributorship	#5a	Names, affiliations, and roles of protocol contributors	16
Roles and responsibilities:	#5b	Name and contact information for the trial sponsor	2

For peer review only

sponsor contact information			
Roles and responsibilities: sponsor and funder	#5c	Role of study sponsor and funders, if any, in study design; collection, management, analysis, and interpretation of data; writing of the report; and the decision to submit the report for publication, including whether they will have ultimate authority over any of these activities	17
Roles and responsibilities: committees	#5d	Composition, roles, and responsibilities of the coordinating centre, steering committee, endpoint adjudication committee, data management team, and other individuals or groups overseeing the trial, if applicable (see Item 21a for data monitoring committee)	16
Background and rationale	#6a	Description of research question and justification for undertaking the trial, including summary of relevant studies (published and unpublished) examining benefits and harms for each intervention	5
Background and rationale: choice of comparators	#6b	Explanation for choice of comparators	5,6
Objectives	#7	Specific objectives or hypotheses	7
Trial design	#8	Description of trial design including type of trial (eg, parallel group, crossover, factorial, single group), allocation ratio, and framework (eg, superiority, equivalence, non-inferiority, exploratory)	5
Study setting	#9	Description of study settings (eg, community clinic, academic hospital) and list of countries where data will be collected. Reference to where list of study sites can be obtained	8
Eligibility criteria	#10	Inclusion and exclusion criteria for participants. If applicable, eligibility criteria for study centres and individuals who will perform the interventions (eg, surgeons, psychotherapists)	9
Interventions: description	#11a	Interventions for each group with sufficient detail to allow replication, including how and when they will be administered	10
Interventions: modifications	#11b	Criteria for discontinuing or modifying allocated interventions for a given trial participant (eg, drug dose change in response to harms, participant request, or improving / worsening disease)	12
Interventions: adherance	#11c	Strategies to improve adherence to intervention protocols, and any procedures for monitoring adherence (eg, drug tablet return;	10,11

For peer review only

		laboratory tests)	
Interventions: concomitant care	#11d	Relevant concomitant care and interventions that are permitted or prohibited during the trial	9
Outcomes	#12	Primary, secondary, and other outcomes, including the specific measurement variable (eg, systolic blood pressure), analysis metric (eg, change from baseline, final value, time to event), method of aggregation (eg, median, proportion), and time point for each outcome. Explanation of the clinical relevance of chosen efficacy and harm outcomes is strongly recommended	11,12
Participant timeline	#13	Time schedule of enrolment, interventions (including any run-ins and washouts), assessments, and visits for participants. A schematic diagram is highly recommended (see Figure)	11
Sample size	#14	Estimated number of participants needed to achieve study objectives and how it was determined, including clinical and statistical assumptions supporting any sample size calculations	13,14
Recruitment	#15	Strategies for achieving adequate participant enrolment to reach target sample size	8
Allocation: sequence generation	#16a	Method of generating the allocation sequence (eg, computer-generated random numbers), and list of any factors for stratification. To reduce predictability of a random sequence, details of any planned restriction (eg, blocking) should be provided in a separate document that is unavailable to those who enrol participants or assign interventions	10,11
Allocation concealment mechanism	#16b	Mechanism of implementing the allocation sequence (eg, central telephone; sequentially numbered, opaque, sealed envelopes), describing any steps to conceal the sequence until interventions are assigned	10,11
Allocation: implementation	#16c	Who will generate the allocation sequence, who will enrol participants, and who will assign participants to interventions	10,11
Blinding (masking)	#17a	Who will be blinded after assignment to interventions (eg, trial participants, care providers, outcome assessors, data analysts), and how	11
Blinding (masking): emergency	#17b	If blinded, circumstances under which unblinding is permissible, and procedure for revealing a participant's allocated intervention during	11

For peer review only

unblinding		the trial	
Data collection plan	#18a	Plans for assessment and collection of outcome, baseline, and other trial data, including any related processes to promote data quality (eg, duplicate measurements, training of assessors) and a description of study instruments (eg, questionnaires, laboratory tests) along with their reliability and validity, if known. Reference to where data collection forms can be found, if not in the protocol	11,12
Data collection plan: retention	#18b	Plans to promote participant retention and complete follow-up, including list of any outcome data to be collected for participants who discontinue or deviate from intervention protocols	10,11
Data management	#19	Plans for data entry, coding, security, and storage, including any related processes to promote data quality (eg, double data entry; range checks for data values). Reference to where details of data management procedures can be found, if not in the protocol	12
Statistics: outcomes	#20a	Statistical methods for analysing primary and secondary outcomes. Reference to where other details of the statistical analysis plan can be found, if not in the protocol	13,14
Statistics: additional analyses	#20b	Methods for any additional analyses (eg, subgroup and adjusted analyses)	14
Statistics: analysis population and missing data	#20c	Definition of analysis population relating to protocol non-adherence (eg, as randomised analysis), and any statistical methods to handle missing data (eg, multiple imputation)	13,14
Data monitoring: formal committee	#21a	Composition of data monitoring committee (DMC); summary of its role and reporting structure; statement of whether it is independent from the sponsor and competing interests; and reference to where further details about its charter can be found, if not in the protocol. Alternatively, an explanation of why a DMC is not needed	16
Data monitoring: interim analysis	#21b	Description of any interim analyses and stopping guidelines, including who will have access to these interim results and make the final decision to terminate the trial	16
Harms	#22	Plans for collecting, assessing, reporting, and managing solicited and spontaneously reported adverse events and other unintended effects of trial interventions or trial conduct	16
Auditing	#23	Frequency and procedures for auditing trial conduct, if any, and	n/a

For peer review only

		whether the process will be independent from investigators and the sponsor	
Research ethics approval	#24	Plans for seeking research ethics committee / institutional review board (REC / IRB) approval	16
Protocol amendments	#25	Plans for communicating important protocol modifications (eg, changes to eligibility criteria, outcomes, analyses) to relevant parties (eg, investigators, REC / IRBs, trial participants, trial registries, journals, regulators)	16
Consent or assent	#26a	Who will obtain informed consent or assent from potential trial participants or authorised surrogates, and how (see Item 32)	8
Consent or assent: ancillary studies	#26b	Additional consent provisions for collection and use of participant data and biological specimens in ancillary studies, if applicable	n/a
Confidentiality	#27	How personal information about potential and enrolled participants will be collected, shared, and maintained in order to protect confidentiality before, during, and after the trial	12
Declaration of interests	#28	Financial and other competing interests for principal investigators for the overall trial and each study site	16
Data access	#29	Statement of who will have access to the final trial dataset, and disclosure of contractual agreements that limit such access for investigators	16
Ancillary and post trial care	#30	Provisions, if any, for ancillary and post-trial care, and for compensation to those who suffer harm from trial participation	n/a
Dissemination policy: trial results	#31a	Plans for investigators and sponsor to communicate trial results to participants, healthcare professionals, the public, and other relevant groups (eg, via publication, reporting in results databases, or other data sharing arrangements), including any publication restrictions	15
Dissemination policy: authorship	#31b	Authorship eligibility guidelines and any intended use of professional writers	n/a
Dissemination policy: reproducible research	#31c	Plans, if any, for granting public access to the full protocol, participant-level dataset, and statistical code	n/a
Informed consent materials	#32	Model consent form and other related documentation given to participants and authorised surrogates	n/a

For peer review only

Biological specimens	#33	Plans for collection, laboratory evaluation, and storage of biological specimens for genetic or molecular analysis in the current trial and for future use in ancillary studies, if applicable	n/a

The SPIRIT checklist is distributed under the terms of the Creative Commons Attribution License CC-BY-ND 3.0. This checklist was completed on 08. November 2018 using http://www.goodreports.org/, a tool made by the EQUATOR Network in collaboration with Penelope.ai

For peer review only
